# The NEON (Nerve rEpair Or Not) trial: a randomized controlled trial of microsurgical repair *versus* nerve alignment for digital nerve injury

**DOI:** 10.1093/bjs/znaf174

**Published:** 2025-09-04

**Authors:** Justin C R Wormald, Matthew D Gardiner, Christina Jerosch-Herold, Jonathan Cook, Rafael Pinedo Villanueva, Ciaron O’Hanlon, Naomi Vides, Gianluca Fabiano, Scott Parsons, Loretta Davies, Heidi Fletcher, Molly Glaze, Cushla Cooper, Dominic Power, Abhilash Jain, David Beard, NEON study group, James Chan, Adam Sierakowski, Clare Langley, Robert Pearl, Rebecca Dunlop, Duncan Avis, Chris Bainbridge, Dominic Furniss, Kshemendra Senarath-Yapa, Susan Stephenson, Nicholas Sheppard, Sarah Kettle, Richard Baker, Oliver Stone, Joshua Totty, Patrick Goon, Alistair Phillips, Rory Norris, Kirti Garude, Timothy Hems, Lauren Uppal, Martina Vitaglione, Anna Beckwith, Natalie Fox, Karen Cranmer, Anna Selby, Sandra Owdziej, Joanna Greenway, Harjoat Riyat, Jiaxin Wen, Linda Tozer, Jamie A Mawhinney, Stephanie Ivie, Pennylouise Hever, Elizabeth Wilson, Donna Kennedy, Caitriona Grace, Adam Misky, Stuart Watson, Tessa Sewdin, Maria Mestre, Soma Farag, Kathryn Lewis, Bipanjit Puar, Claire Sethu, Akira Wiberg, Isabel Teo, Terry-Ann Curran, Oliver Jones, Tracey Shewan, Cassandra Honeywell, Suzanne Dean, Eve Fletcher, Mate Zabaglo, Ana Dias, Muhammad Haruna, Kerry Anderson, Geraldine Hambrook, Bobbie Sanghera, Katarina Manso, Judith Abrams, Iona Burn, Rebecca Shirley, Richard Goodall, Mina Ip, Alistair Reed, Zoe Avent, Maria Chicco, Peter Cronbach, David Cussons, Alexander Baldwin, Danielle Johnson, Ken Wong, Kerri McGowan, Marc Atkinson, Joana Da Rocha, Mohamed Sheikh, Abigail Owen, Angie Dempster, Albina Morozova, Agnes Lagare

**Affiliations:** Surgical Interventional Trials Unit (SITU), Nuffield Department of Orthopaedics, Rheumatology and Musculoskeletal Sciences, University of Oxford, Oxford, UK; Department of Plastic and Reconstructive Surgery, Oxford University Healthcare NHS Foundation Trust, Oxford, UK; Surgical Interventional Trials Unit (SITU), Nuffield Department of Orthopaedics, Rheumatology and Musculoskeletal Sciences, University of Oxford, Oxford, UK; Department of Plastic and Reconstructive Surgery, Frimley Health NHS Foundation Trust, Slough, UK; Rehabilitation Research, School of Health Sciences, University of East Anglia, Norwich, UK; Surgical Interventional Trials Unit (SITU), Nuffield Department of Orthopaedics, Rheumatology and Musculoskeletal Sciences, University of Oxford, Oxford, UK; Surgical Interventional Trials Unit (SITU), Nuffield Department of Orthopaedics, Rheumatology and Musculoskeletal Sciences, University of Oxford, Oxford, UK; Surgical Interventional Trials Unit (SITU), Nuffield Department of Orthopaedics, Rheumatology and Musculoskeletal Sciences, University of Oxford, Oxford, UK; Surgical Interventional Trials Unit (SITU), Nuffield Department of Orthopaedics, Rheumatology and Musculoskeletal Sciences, University of Oxford, Oxford, UK; Surgical Interventional Trials Unit (SITU), Nuffield Department of Orthopaedics, Rheumatology and Musculoskeletal Sciences, University of Oxford, Oxford, UK; Surgical Interventional Trials Unit (SITU), Nuffield Department of Orthopaedics, Rheumatology and Musculoskeletal Sciences, University of Oxford, Oxford, UK; Surgical Interventional Trials Unit (SITU), Nuffield Department of Orthopaedics, Rheumatology and Musculoskeletal Sciences, University of Oxford, Oxford, UK; Surgical Interventional Trials Unit (SITU), Nuffield Department of Orthopaedics, Rheumatology and Musculoskeletal Sciences, University of Oxford, Oxford, UK; Surgical Interventional Trials Unit (SITU), Nuffield Department of Orthopaedics, Rheumatology and Musculoskeletal Sciences, University of Oxford, Oxford, UK; Surgical Interventional Trials Unit (SITU), Nuffield Department of Orthopaedics, Rheumatology and Musculoskeletal Sciences, University of Oxford, Oxford, UK; Birmingham Hand Centre, Queen Elizabeth Hospital, Birmingham, UK; Surgical Interventional Trials Unit (SITU), Nuffield Department of Orthopaedics, Rheumatology and Musculoskeletal Sciences, University of Oxford, Oxford, UK; Department of Plastic and Reconstructive Surgery, Imperial College Healthcare NHS Trust, London, UK; Surgical Interventional Trials Unit (SITU), Nuffield Department of Orthopaedics, Rheumatology and Musculoskeletal Sciences, University of Oxford, Oxford, UK; NHMRC Clinical Trials Centre, Faculty of Medicine & Health, University of Sydney, Sydney, New South Wales, Australia

## Abstract

**Background:**

Digital nerves provide sensibility to the fingers. They are commonly injured through accidental sharp laceration. The aim of the NEON (Nerve rEpair Or Not) study was to investigate whether microsurgical suture repair of lacerated digital nerves is superior to nerve alignment alone without suture repair.

**Methods:**

A two-arm, parallel group, double-blind, multicentre RCT was undertaken over 2 years. Participants with suspected unilateral digital nerve injury underwent surgical exploration and were randomized to microsurgical suture repair or nerve alignment alone. The primary outcome was the Impact of Hand Nerve Disorders (I-HaND v2) patient-reported outcome measure (PROM) at 12 months post-randomization. Secondary outcomes assessed were: objective neurosensory and functional recovery; health-related quality of life to examine cost-effectiveness; complications of surgery and clinically problematic neuroma rates (Elliot score). Both participants and assessors were blind to allocation.

**Results:**

A total of 122 adults were randomized to microsurgical suture repair (*n* = 61) or nerve alignment alone (*n* = 61). Primary outcome data using the I-HaND (v2) were available for 106 participants (87%) at 12 months. There were no statistically significant differences in I-HaND scores at all time points, including the 12-month primary end point (15.9 *versus* 20.2, *P* = 0.09; 95% c.i. [−0.9, 10.8]). There were also no differences in all secondary outcome measures, including Patient Evaluation Measure and EQ-5D-5L scores at 12 months. Complications were similar at 6 weeks and 12 months. The trial was closed early by the funder owing to slow recruitment and did not reach the intended sample size.

**Conclusion:**

Based on the available data from the NEON trial, there is no evidence to support the beneficial effect of suture repair over nerve alignment alone for isolated digital nerve injury. This multicentre RCT can be used to inform future trials, inform patients and guide clinical practice.

**Funding:**

NIHR Health Technology Assessment (NIHR127807–18/37).

**Trial registration number:**

ISRCTN16211574

## Teaser

This randomized comparison of microsurgical suture repair *versus* nerve alignment alone for digital nerve injury did not detect any differences between treatments at 12 months. This is the first study of its kind. It can be used for clinical decision-making and to guide future research.

## Introduction

Digital nerve injuries of the finger are the most common nerve injury treated surgically in the UK and represent 9.0% of all hand injuries^1,2^. Based on an analysis of Hospital Episode Statistics (HES) data in England and Wales, approximately 3000 digital nerve repairs are undertaken in the UK annually^1,2^. There are about 28 000 cases per year in Europe and 18 600 per year in the USA^3,4^. Usual treatment for digital nerve injuries involves specialist referral and direct end-to-end suture repair of the nerve ends, with an operating microscope or loupe magnification, in an operating room. The goals of digital nerve injury treatment are to restore sensibility and prevent cold intolerance and neuroma^5,6^. The rationale for suture repair is that it gives patients the best chance of sensory recovery and reduces the risk of neuroma formation.

A systematic review in 2019 analysed all studies of suture repair of digital nerve injury, compared where possible, with injuries that were not repaired^7^. There were no RCTs and all included studies were small single-centre observational studies. All studies were case series of between 15 and 110 nerve injuries, with heterogeneous patient, injury, and treatment characteristics. None of the studies used outcome measures that reflected the functional impact of sensory impairment due to patients’ adapting behaviour and assessor skill^6^. Only two studies had comparisons between repaired and non-repaired digital nerves, although these were non-randomized and at high risk of bias^8,9^.

The British Society for Surgery of the Hand (BSSH) identified the repair of digital nerves as a health resource topic to study, confirmed through a James Lind Alliance Priority Setting Exercise^10^. Responses from a survey of over 140 surgeons and therapists prior to the conduct of the study confirmed the existence of community equipoise. Although some respondents stated that suture repair is effective and essential, a considerable number were uncertain of effectiveness and confirmed the need for and willingness to engage with such a trial^11^.

The aim of the NEON (Nerve rEpair Or Not) study was to determine whether suture repair of a digital nerve is effective by comparing two interventions: surgical exploration, nerve alignment, and microsurgical suture repair *versus* surgical exploration and nerve alignment alone. Given the lack of current evidence, the high frequency of the procedure and the lack of resources in the NHS, a robust trial was required to address the surgical dogma that surrounds this injury and its management.

## Methods

**Fig. 1:**
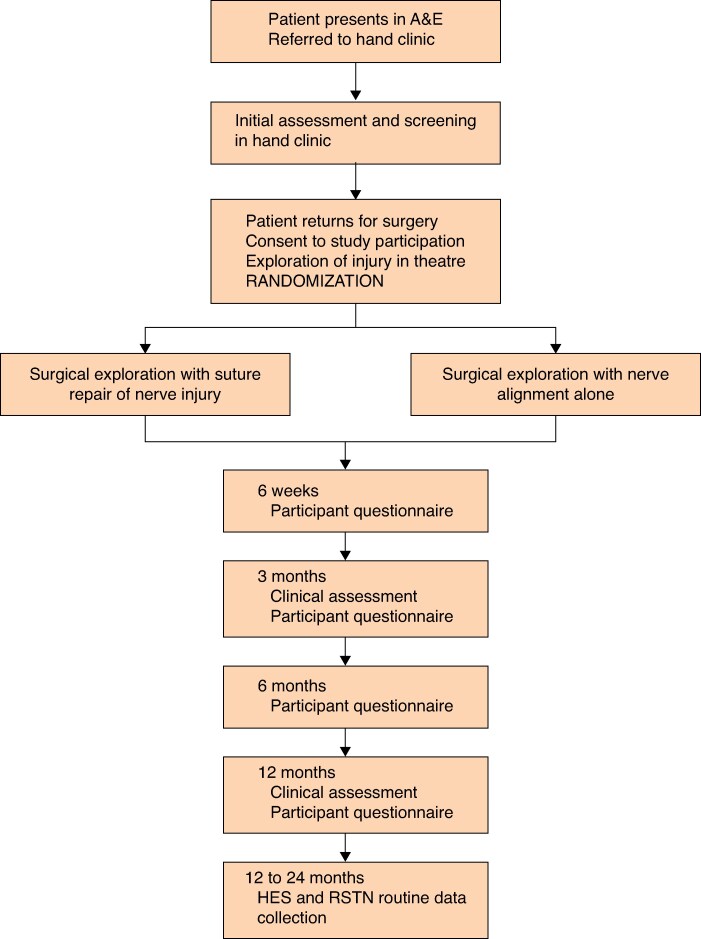
Participant flow diagram for the NEON study

The NEON study (Ethics ref: 20/SC/0018) was designed as a multicentre RCT (ISRCTN16211574) with 1:1 allocation and internal pilot phase. It was planned for participants to be recruited from 25 NHS hospitals across the UK (*Fig. 1*)^12^.

RSTN, Reconstructive Surgery Trials Network.

### Primary objectives

To ascertain the effectiveness of nerve alignment and suture repair *versus* nerve alignment alone for patients with digital nerve injuries, using the Impact of Hand Nerve Disorders (I-HaND v2) patient-reported outcome measure (PROM), at 6 weeks, 3 months, and 12 months post-randomization^13^.

### Secondary objectives

- To further assess and compare neurosensory and functional recovery and health-related quality of life in the two treatment arms.
- To compare the cost-effectiveness of the two treatment arms.
- To compare complications of surgery and clinically problematic neuroma rates in the two treatment arms.

### Study participants

Patients 18 years and over presenting with a single unilateral digital nerve injury appropriate for surgical repair were considered for the NEON study.

#### Inclusion criteria

Patients aged 18 years and above with a suspected complete single digital nerve laceration in any single digit, including thumb and little finger, appropriate for surgical exploration and repair were included.

#### Exclusion criteria

**Fig. 2:**
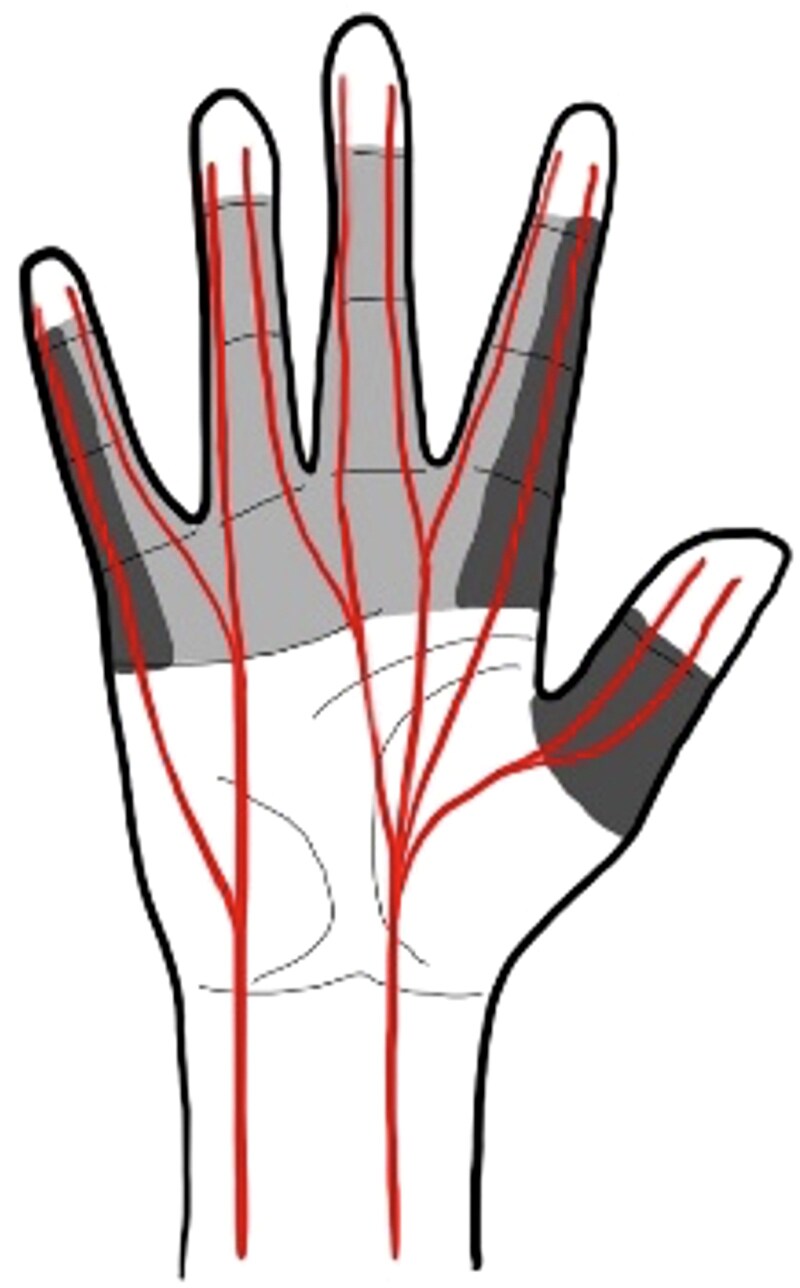
Schematic diagram showing digital nerve anatomy Grey shading denotes zone of injury for inclusion into the trial. Dark shading denotes zone of injury in the ‘critical zones’, a minimization factor in the study.

Patients were excluded if they had: bilateral injuries (that is both radial and ulnar digital nerves injured), lacerations outside the region between distal palmar crease and distal interphalangeal joint, closed injuries, infected wounds, injuries in which a significant nerve gap existed (precluding direct tension free surgical end–end repair), non-isolated or multilevel injury (that is common digital/wrist nerve injury, fracture). They were also excluded if they were unable to give consent or comply with study follow-up procedures, if surgery took place later than 10 days post-injury, and if they had no or an incomplete digital nerve injury on surgical exploration. Concomitant flexor tendon injuries and lacerations in ‘critical zones’ (*Fig. 2*) were not excluded as these are often features of this injury type. These were minimization factors in the randomization to balance the number of each in the two groups.

### Recruitment, randomization, and blinding

Potential participants were approached and screened for eligibility at specialist hand clinics following presentation at Emergency Departments (ED) or minor injury units. Following consent, participants underwent surgery. A further eligibility assessment was conducted intraoperatively once wound exploration confirmed digital nerve division amenable to repair. Randomization followed a 1:1 allocation ratio and use of a minimization algorithm. During wound exploration, the surgeon identified whether the injury involved flexor tendon damage or affected a ‘critical zone’ (*Fig. 2*). Randomization was minimized according to these two factors, as well as by study site. Randomization was performed using a web-based automated computer-generated system. The research team at each site conducted the randomization via secure logins to the web-based system. For those patients having surgery under local anaesthetic, the randomization was done without revealing the intended treatment to the patient, thus protecting blinding. Participants and follow-up assessors were blinded to the randomized allocation. It was not possible to blind theatre staff. Follow-up assessors were blinded throughout the study to ensure objective sensory measurements. Participants in both arms received identical follow-up regimens.

### Interventions

The procedures were undertaken as per NHS practice, in a sterile surgical environment, by surgeons based in specialist hand surgery units. Procedures were done either as a day case (>95%) or as an inpatient, and under either general or local anaesthesia (87%) as per clinical judgement.

#### Surgical exploration, nerve alignment, and suture repair

The laceration was surgically explored, debrided, and irrigated as per standard practice. Participants allocated to the suture group then had the cut nerve ends aligned and repaired using end-to-end epineural microsurgical sutures. This was performed under either the operating microscope or with loupe magnification. Surgeons were permitted to choose their preferred magnification and suture material, which was recorded.

#### Surgical exploration and nerve alignment alone

The laceration was surgically explored, debrided, and irrigated as per standard practice. After exploration and washout participants allocated to the nerve alignment alone group had the cut nerve ends aligned but not sutured.

#### Completion of surgical procedure

Any associated injuries, such as flexor tendon injury, were repaired, and the wound closed as per standard care. Dressings were applied as per surgeon preference, and depended on the extent of the injury, subsequent dissection, and other associated injuries.

### Postoperative rehabilitation

Simple (isolated) digital nerve injuries (without concomitant tendon or other tissue injury) are not routinely referred for hand therapy. For the purposes of this study, all participants with these simple injuries were provided with a standardized advice sheet and a sensory relearning advice sheet, given at discharge. Participants with concomitant injuries were referred to hand therapy for rehabilitation as per standard practice. Hand therapists issued the same sensory relearning advice sheet when deemed safe for the participant to do the exercises without compromising tendon healing.

### Outcome measurement

The primary outcome measure was clinical effectiveness of microsurgical nerve repair measured by the I-HaND PROM at 12 months post-randomization^13^. Participant questionnaires were completed at 6 weeks and 3, 6, and 12 months post-randomization and included the primary outcome measure I-HaND v2 and other secondary outcome measures listed below^13,14^. The I-HaND Is the only nerve-specific, hand-specific PROM that has been developed using contemporary psychometric properties in accordance with ISOQOL and COSMIN standards^13^. It was developed using stringent standards for PROM development including in-depth qualitative research and cognitive interviewing with hand nerve injury patients. Version 2.0 has 32 items and covers symptoms, physical difficulties, and feeling about their hands, pain, work, and ability to undertake activities and work. It has demonstrated high content validity, excellent test–retest reliability, construct validity, and is responsive to change. The I-HaND is the most relevant PROM for this patient population compared to existing region-specific PROMs developed for hand and upper limb trauma or musculoskeletal conditions. This was further confirmed during patient and public involvement (PPI) consultation where patients showed a clear preference for I-HaND over other PROMs.

Secondary outcome measures included neurosensory and functional recovery measured at 3 and 12 months post-randomization by

(a) Hand Health Profile of the Patient Evaluation Measure (13—PEM)^15^; (b) EQ-5D-5L index and EQ-VAS^14^; (c) static two-point discrimination test (2PD)^16^; (d) tactile gnosis using Shape/Texture Identification (STI) test; and touch thresholds using Weinstein Enhanced Sensory Test (WEST) monofilaments^17^.
Cost-effectiveness of microsurgical nerve repair, measured by healthcare resource use questionnaires and EQ-5D-5L index at 3 and 12 months post-randomization.
Complications of surgery and clinically problematic neuroma rates measured at 3 and 12 months post-randomization by: (a) patient-reported complications; (b) clinical assessment (including Elliot score); (c) complications and further procedures in medical records; (d) complications and further procedures based on medical records and routine data at 24 months post-randomization.

The 6-month questionnaire included the EQ-5D-5L and health resource use questionnaire only^18^. Clinical assessments were undertaken at 3 and 12 months post-randomization, either in person or remotely over the phone. It was planned for this assessment to be conducted by research nurses or research therapists unaware of the allocation and not involved in routine patient care. Medical note checks were performed at the 3-month follow-up time point, including recording of any postoperative hand therapy sessions. At 3 and 12 months post-randomization, any complications related to the participant's digital nerve injury documented in the medical notes were also collated. The clinical assessment at 3 and 12 months post-randomization comprised of checking for two complications of surgery: neuroma captured by the Elliot score and sensitivity to cold^15^. The Elliot score is a set of five questions about pain covering spontaneous pain (basal and spikes), pressure pain, movement pain and hypersensitivity. Cold intolerance was assessed as one of three levels.

### Sample size

The sample size was based upon I-HaND data for patients with hand nerve conditions and sample of digital nerve patients, the standard deviation was as high as 21 points^19^. Previous work in a similar measure suggests 7 would be a clinically important difference (0.3 standardized effect size)^19–22^. To detect a target mean difference of 7 points in the I-HaND (v2) with a standard deviation of 21, 2-sided 5% significance level, and 90% statistical power, 191 participants were required per group (382 overall)^13,19,20^. Based on the team's experience of other digital nerve studies and hand trials, 20% missing data was allowed for, giving an overall target of 478.

### Statistical analysis

All participants were grouped according to their randomly allocated group (‘as randomized’) or intention to treat (‘ITT’). All principal analyses were performed at the 2-sided 5% significance level. The primary outcome measure (I-HaND v2) was compared using a linear regression model with fixed effects of treatment arm, indication of critical zone, indication of tendon injury and adjustment for the site using cluster robust variance. A secondary unadjusted analysis was also carried out by an independent *t* test. The primary outcome was summarized by treatment groups and displayed visually using boxplots. For the secondary outcome measures (Hand Health Profile of PEM, Static 2PD, STI, WEST, Patient Global Rating of Sensation (GROS), EQ-5D-5L and EQ-VAS) differences between treatment groups were compared in the same way as the primary analysis, with an adjusted linear regression model and additional *t* test. This was also performed on the ‘as randomized’ population. Complications were summarized by treatment group at 6 weeks post-randomization. Cold intolerance scores and Elliot scores at 3 and 12 months were summarized. Cold intolerance was analysed using logistic regression (comparing any pain (1 or 2) with no pain (0)), adjusting for minimization factors. Elliot score was analysed using a linear regression model as in the primary analysis. A health-related quality of life and healthcare resource use analysis was carried out, which included an exploratory descriptive analysis of EQ-5D-5L and EQ-VAS at 6 weeks and 3, 6, and 12 months follow-up and healthcare resource use at 3, 6, and 12 months follow-up based on participant completed questionnaires. Overall EQ-5D scores were calculated by mapping the 5L responses onto the 3L value set as per National Institute of Health and Care Excellence (NICE) recommendation^23,24^. Due to the early stopping of the trial and data available, no full cost analysis for the trial was conducted.

### Study within a trial

A prospective study within a trial (SWAT), embedded within the NEON trial, investigated the diagnostic accuracy of physical examination in identifying patients with complete digital nerve injuries. The study report for this will be published separately.

## Results

**Fig. 3:**
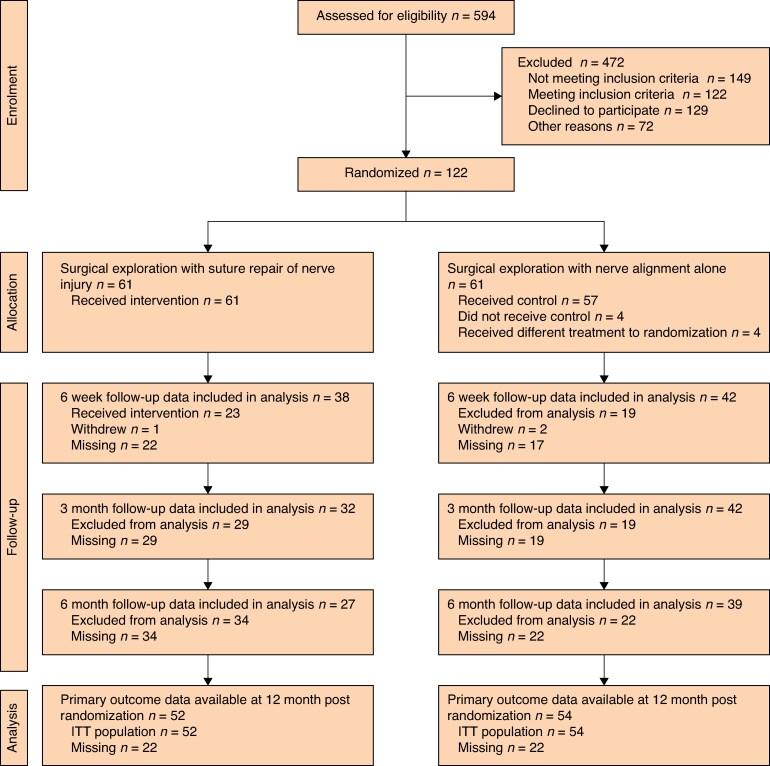
CONSORT diagram

The study aimed to open 25 sites. By November 2022, when the trial was closed to recruitment, 17 sites had opened to recruitment. The first patient was registered on 20 September 2020. Recruitment was stopped by the funder on 11 November 2022. The final study visit for the final patient was on 23 November 2023. The flow of participants through all stages of the trial—eligibility, registration, allocation, and analysis—is presented in the CONSORT diagram (*Fig. 3*)^25^. Across the study period, 594 were assessed for screening and 122 were randomized, 61 into each of the two treatment groups. The primary outcome, I-HaND (v2) at 12 months, was available for 106 (87%) participants.

### Randomization and baseline characteristics

Detailed screening data and reasons for exclusion are presented in *Table 1*. Baseline randomization minimization factors of site, flexor tendon damage, and critical zone affected in the hand injury are summarized in *Table S1* and demonstrate good balance between treatment groups. *Table 2* shows information on the baseline characteristic comparability by allocated treatment arm.

**Table 1:** Reasons for exclusion (20 September 2020–11 November 2022)

Category	N
Total screened	594
Total randomized	122
Total excluded	472
**Not meeting inclusion criteria**	149
Not aged 18 and above	0
Not suspected complete digital nerve laceration in any single digit	149
**Meeting exclusion criteria**	122
Bilateral injury (that is both radial and ulnar digital nerves)	5
Laceration outside the region between distal palmar crease and distal interphalangeal joint	35
Closed injury	0
Infected wounds	12
Injuries in which a significant nerve gap exists which would preclude direct tension-free surgical end–end repair	4
Non-isolated or multilevel injury (that is common digital/wrist nerve injury, fracture)	24
Unable to give consent	11
Inability to comply with study follow-up procedures	22
Date of surgery later than 10 days after injury	9
Digital nerve incomplete or not present	0
Total ineligible	271
**Declined to participate**	129
Patient did not want to be randomized	51
Patient expressed preference for non-surgical treatment	4
Patient did not give a reason	19
Other	55
**Eligible but not approached for consent**	33
No research staff available	7
Clinical decision	13
Other	13
Reason for exclusion missing	30

**Table 2:** Baseline characteristics of participants—split by treatment group and overall

	Suture repair (*n* = 61)	Nerve alignment alone (*n* = 61)	Total (*n* = 122)
Age*	42.33(13.59), 42.00 [32.00, 53.00], {18.00, 77.00}	42.02(17.51), 43.00 [26.00, 54.00], {18.00, 78.00}	42.17(15.61), 42.50 [28.25, 53.00], {18.00, 78.00}
**Sex†**			
Male	41 (67.21)	41 (67.21)	82 (67.21)
Female	20 (32.79)	20 (32.79)	40 (32.79)
**Smoking†**			
No	46 (76.67)	47 (79.66)	93 (76.23)
Yes	14 (23.33)	12 (20.34)	26 (21.31)
**Diabetes†**			
Yes	3 (4.92)	1 (1.67)	4 (3.28)
No	58 (95.08)	59 (98.33)	117 (95.90)
**Injury details—hand†**			
Left	41 (67.21)	40 (65.57)	81 (66.39)
Right	20 (32.79)	21 (34.43)	41 (33.61)
**Injury details—finger†**			
Thumb	8 (13.11)	10 (16.39)	18 (14.75)
Index	24 (39.34)	20 (32.79)	44 (36.07)
Middle	10 (16.39)	8 (13.11)	18 (14.75)
Ring	4 (6.56)	4 (6.56)	8 (6.56)
Little	15 (24.59)	19 (31.15)	34 (27.87)
**Injury details—digital nerve†**			
Radial digital	36 (59.02)	37 (60.66)	73 (59.84)
Ulnar digital	25 (40.98)	24 (39.34)	49 (40.16)
**Type of anaesthetic†**			
General	5 (8.20)	7 (11.48)	12 (9.84)
Local	55 (90.16)	51 (83.61)	106 (86.89)
Both	1 (1.64)	3 (4.92)	4 (3.28)

*Summaries are mean(s.d.), Median [i.q.r.], {range}. †Summaries are *n* (%).

### Compliance

Of the 61 participants randomized to suture repair, 61 (100%) received the intervention as planned, as shown in *Table S2*. For the nerve alignment alone group, 57 of the 61 randomized participants received the intervention as planned. In four of the nerve alignment alone operations, the surgeon opted to repair the nerve with sutures after visual inspection of the injury. Surgery was conducted on the day of randomization except for one patient who had surgery 2 days after randomization. The majority (73%) of operations across both groups were performed by either consultants (26%) or specialist registrars (47%). Indeed, there was a higher proportion of consultants in the suture repair group (31%) than in the alignment alone group (21%). All operations were performed within the NHS, by NHS surgeons who have been trained in a nationalized, standardized system. This was a multicentre pragmatic study and so the results reflect everyday practice within the NHS. This, in addition to robust randomization, means that there are no systematic differences between the two intervention groups.

Further details of the surgery received, for example time in theatre, are given in *Tables S3*, *S4*. Complications experienced during surgery are summarized in *Table S5*.

### Retention

*Table S6* provides a summary of the completion of follow-up questionnaires, with numbers received and percentage received out of total expected. The availability of primary outcome (I-HaND v2) for each questionnaire time point was 66% (6 weeks), 61% (3 months), and 87% (12 months). Of the 121 participants randomized, seven withdrew from the trial, three in the suture repair arm and four in the nerve alignment alone arm. The details of withdrawals by each time point by treatment group are summarized in *Table S7*.

### Primary outcome: sensory hand function

The nerve alignment alone group (*n* = 54) had mean(s.d.) I-HaND scores at 12 months of 15.90(12.32), compared to 20.18(16.61) for the suture repair group (*n* = 52) group, with lower scores indicating better function. This observation was not statistically significant (adjusted difference 4.94, 95% c.i. (−0.93, 10.82), *P* = 0.09 and unadjusted difference 4.28 95% c.i. (−1.34, 9.89) *P* = 0.13) either due to no true difference or underpowering. A similar difference was observed for the 3- and 6-month time points (*Table 3*).

**Table 3:** Results of I-HaND (v2) score by treatment groups—ITT population

	Suture repair (*n* = 61)*	Nerve alignment alone (*n* = 61)*	Adjusted difference (95% c.i.)†	*P*	Unadjusted difference (95% c.i.)‡	*P*
6 weeks	*n* = 39, 32.22(18.88)	*n* = 42, 25.09(14.76)	7.27 (−1.4, 15.94)	0.093	7.13 (−0.33, 14.6)	0.061
3 months	*n* = 32, 25.41(13.45)	*n* = 42, 21.10(15.28)	4.41 (−2.75, 11.57)	0.206	4.31 (−2.48, 11.11)	0.21
12 months	*n* = 52, 20.18(16.61)	*n* = 54, 15.90(12.32)	4.94 (−0.93, 10.82)	0.092	4.28 (−1.34, 9.89)	0.134

I-HaND (v2): A higher score indicates greater disability (range 32–160 points). *Impact of Hand Nerve Disorder (I-HaND v2). Summaries are mean(s.d.). †Linear regression model, adjusted for minimization factors (critical or non-critical zone, associated tendon injury). Study site was accounted for using cluster robust variance. ‡Unadjusted analysis was an independent *t* test.

### Secondary outcomes

There was no difference in PEM scores at 12 months (*n* = 102, *P* = 0.26; adjusted difference 4.03, 95% c.i. (−3.4, 11.4), *Table S9*). There were no statistically significant differences in EQ-5D and EQ-VAS between the groups (*n* = 107, *P* = 0.40, *P* = 0.07, *Table S10*). Patient GROS data showed no difference between groups at 12 months (*n* = 46, *P* = 0.83, *Table S11*). WEST scores were available for a small subgroup of participants at 12 months and were not different (*n* = 27, *P* = 0.07; adjusted difference 4.66, 95% c.i. (1.0, 26.6), *Table S12*). Static 2PD was only reported by 11 suture repair participants and 12 nerve alignment alone participants. At 12 months, the suture repair group had slightly more accurate 2PD compared to the nerve alignment group (9.8 mm *versus* 11.2 mm, adjusted difference −1.7 mm, 95% c.i. (3.21, −0.2), *P* = 0.03, *Table S13*). At 12 months there was no difference in STI between the groups (*n* = 27, *P* = 0.62, *Table S14*).

### Complications

Patient-reported complications, including scar sensitivity, delayed wound healing, and persistent ongoing pain, were similar in both groups at 6 weeks (*n* = 26 suture repair *versus n* = 24 nerve alignment alone, *Table S15*). Details on any complications and any further procedures reported in medical records were similar in both groups at 3 months (*n* = 18 repaired *versus n* = 20 non-repaired) and 12 months (*n* = 13 repaired *versus n* = 9 non-repaired) post-randomization are summarized in *Table S16*.

#### Elliot score and sensitivity to cold

There were no statistically significant differences in Elliot scores or cold intolerance at 3 or 12 months (*Tables S17–20*). At 12 months, 4 (27%) participants in the suture repair group (*n* = 15) had an Elliot score indicating suspected neuroma. In the nerve alignment alone (*n* = 12) this was the case for 1 participant (2%), who underwent further surgery for neuroma by 12 months.

### Health resource use

#### Secondary care

Two participants in the suture repair group reported inpatient hospitalizations for reoperations (not for neuroma) at 3 months (*n* = 33). Between 6 months and 12 months, a further two participants in the suture repair group reported an admission (*n* = 56). None of the participants in the nerve alignment (control) group reported being admitted for a reoperation. The proportion of participants reporting hospital outpatient consultations over the first six months was similar between groups: 4 of 26 (15%, suture repair) *versus* 7 of 37 (19%, nerve alignment alone). During the last 6 months of follow-up, 5 of 56 participants (9%) in the suture repair and 5 of 51 (10%) in the nerve alignment group reported outpatient visits. Consultations were mostly with a therapist and with the hand surgery service for both groups.

#### Primary and community care

One of 26 participants in the suture repair group reported a visit over the first 6 months, and 2 of 55 over the following 6 months. For the nerve alignment alone group, 4 of 36 attended appointments over the first 6 months post-surgery, with 1 of 51 by 12 months. Consultations were mainly with the GP or a community hand therapist.

#### Time off work

Over the first 3 months, 15 of 24 participants (63%) in the suture repair group reported taking time off (mean 30 days). For participants in the nerve alignment alone group, 12 of 26 (46%) took time off during the same period (mean 16 days).

### Missing data

As there were more than 5% missing data at 12 months, as per the analysis plan, sensitivity analyses assessing the impact of missing data was performed. *Table S8* shows the distribution of missing data between treatment groups at 6 weeks, 3 months, and 12 months.

### Safety reporting

There were three serious adverse events (SAEs) in total, one in the suture repair group and two in the nerve alignment alone group, all of which were unrelated to the treatment (*Table S2^1^*).

## Discussion

This randomized comparison of nerve alignment, with or without suture repair, is the largest study of its kind in this common injury. Despite being closed early by the funder, this RCT provides new evidence on the effectiveness of suture repair of digital nerve injury, as well as insights on the design and delivery of future RCTs in hand injury. Although 40% of potentially eligible patients declined to be involved in NEON, the substantial majority were keen to join the trial. Unfortunately, as a result of the COVID-19 pandemic and its sequelae, it was challenging to recruit and open sites for the trial. This resulted in early closure by the funder. There were trends towards at least equivocal scores in the nerve alignment alone group for almost all outcomes and time points. This suggests that nerve alignment without suture repair of the severed nerve was as beneficial, if not more so, in some outcomes, such as hand nerve injury-specific PROM scores. I-HaND scores were lower in the nerve alignment alone group compared to the suture repair group, with all other measures of hand function and health-related quality of life, the same between groups. One participant in the nerve alignment alone group underwent surgery for neuroma, with more participants with potential neuroma in the suture repair group (*n* = 4). The difference in neuroma risk, measured with Elliot scores, was not statistically significant in this study. Other complications were essentially equivocal, including cold intolerance, hypersensitivity, stiffness, and complex regional pain syndroms (CRPS). More participants in the suture repair group had postoperative swelling. These results indicate that suture repair does not confer substantial benefit over nerve alignment alone in digital nerve injury across all hand, nerve ,and generic quality of life outcomes.

To date, as summarized by the systematic review in 2019 and subsequent publications, solely observational, usually retrospective and non-comparative, single-centre studies have comprised the evidence base for management of digital nerve injury^8,9,26–37^. These studies are at high risk of bias, are confounded by both known and unknown variables, and do not provide data that are sufficiently robust to inform practice. The prospective case series from Chow and Ng compared outcomes between nerve repair and non-repair, involving 72 repaired nerves and 36 unrepaired nerves. Of the initial cohort of 132 patients, 47 (36%) were lost to follow-up^8^. As with NEON, Chow and Ng also suggested an improvement in static 2PD in the small, surgically repaired group. None of the patients in the non-repair group elected to undergo secondary surgical intervention, and 94% attained restoration of protective sensibility. Poor retention and high risk of bias mean the data from this single-centre observational study are unreliable^8^. In a more recent non-randomized, single-centre study of 74 patients who underwent suture repair (*n* = 42) or no repair (*n* = 32), digital nerve repair improved static 2PD but did not affect sharp/dull discrimination, warm/cold sensation, VAS subjective satisfaction evaluations or patient satisfaction^9^. This study was similarly at high risk of bias and consequently the results are unreliable. The only consistent finding across these two comparative studies and the NEON trial is the inconsequential improvement in static 2PD in sutured nerves, a finding that has now been demonstrated to not result in improved nerve-related hand function.

During the COVID pandemic the BSSH issued guidelines that isolated digital nerve injuries should not be surgically repaired to reduce risk of COVID based on the preliminary work and equipoise existing for the NEON trial. The results of this policy have not yet been collated and disseminated, but anecdotally we have not seen a substantial rise in patients requiring neuroma excision since the end of the pandemic in the UK. The results of this abbreviated RCT confirm this and further question the effectiveness of suture repair for digital nerves.

This study provides valuable feasibility data for future research, demonstrating that both patients and surgeons are willing to recruit and randomize to such trials. It also shows that we can adequately follow these participants to clinically important end points using validated site- and condition-specific PROMs. This builds on existing feasibility data for hand trauma RCTs reported in the HAWAII feasibility trial, a randomized evaluation of antimicrobial sutures in hand trauma, although the NEON trial achieved better retention at greater time points^38^. Both NEON and POINT, another RCT in hand injury comparing surgery *versus* splinting for phalangeal fractures, were closed early by the funder. Although this means that limited effectiveness data can be drawn on the interventions in question, both provide highly valuable feasibility and operational data on delivering high-quality research in hand trauma^39^. This trial has demonstrated that patients will accept a trial that includes non-suture repair of digital nerve injury.

The trial also had some methodological nuances. It revealed substantial uncertainty amongst the hand surgery community around the benefits of suture repair for digital nerves with some polarized views on the effectiveness of treatment or the need for evaluation^11,40^. The research question was whether to suture repair the nerve, but this was often conflated with whether surgery was needed. From a methodology perspective, addressing community equipoise and obtaining consensus on the research question is clearly an important issue for hand surgery trials and should be considered early in future RCTs. These characteristics have some influence on interpretation below and the need, and direction, of future studies.

In terms of impact, the clinical interpretation of the main findings is not straightforward. On the one hand, the failure to reach the *a priori* sample size does compromise any final conclusions and digital nerve suture repair should certainly not be dismissed based on the NEON study. Nonetheless, the act of suturing a nerve is unlikely to be better than carefully aligning the nerve ends. This raises the question whether a patient needs an operation to explore a suspected nerve injury in the first place. The NEON study did not address this question. Not needing to suture the nerve does not equate to not needing an operation. The act of debriding the wound and aligning the nerve ends may still confer a benefit over leaving the wound and nerve in the injured state. Considering that reinnervation inaccuracy may lead to permanent functional loss, it is logical that nerve alignment would result in good functional recovery observed in this RCT^41^. Equally, the presence of inorganic suture material, along with the physical trauma that placing sutures may incur to the nerve, could result in a local environment that is poorly suited for nerve regeneration^42^. Treatment decisions should remain patient centred, with some patients better suited to repair for a variety of reasons. The study has shown that the choice of suture repair or not should become more considered and judicious.

Despite the suboptimal sample size, the NEON trial is now the largest multicentre and non-industry-funded RCT of surgical intervention for digital nerve injury to date^34,43,44^. The results therefore provide the current best evidence for practice and can be used to inform patients and surgeons and guide clinical practice. Even with an incomplete sample, the NEON study has shown that nerve alignment without suture repair of the severed nerve is a satisfactory intervention and may even be more suitable than the current practice of suture repair for some cases.

## Collaborators

James Chan, Adam Sierakowski, Clare Langley, Robert Pearl, Rebecca Dunlop, Duncan Avis, Chris Bainbridge, Dominic Furniss, Kshemendra Senarath-Yapa, Susan Stephenson, Nicholas Sheppard, Sarah Kettle, Richard Baker, Oliver Stone, Joshua Totty, Patrick Goon, Alistair Phillips, Rory Norris, Kirti Garude, Timothy Hems, Lauren Uppal, Martina Vitaglione, Anna Beckwith, Natalie Fox, Karen Cranmer, Anna Selby, Sandra Owdziej, Joanna Greenway, Harjoat Riyat, Jiaxin Wen, Linda Tozer, Jamie A. Mawhinney, Stephanie Ivie, Pennylouise Hever, Elizabeth Wilson, Donna Kennedy, Caitriona Grace, Adam Misky, Stuart Watson, Tessa Sewdin, Maria Mestre, Soma Farag, Kathryn Lewis, Bipanjit Puar, Claire Sethu, Akira Wiberg, Isabel Teo, Terry-Ann Curran, Oliver Jones, Tracey Shewan, Cassandra Honeywell, Suzanne Dean, Eve Fletcher, Mate Zabaglo, Ana Dias, Muhammad Haruna, Kerry Anderson, Geraldine Hambrook, Bobbie Sanghera, Katarina Manso, Judith Abrams, Iona Burn, Rebecca Shirley, Richard Goodall, Mina Ip, Alistair Reed, Zoe Avent, Maria Chicco, Peter Cronbach, David Cussons, Alexander Baldwin, Danielle Johnson, Ken Wong, Kerri McGowan, Marc Atkinson, Joana Da Rocha, Mohamed Sheikh, Abigail Owen, Angie Dempster, Albina Morozova, Agnes Lagare.

## Supplementary Material

znaf174_Supplementary_Data

## Data Availability

Anonymised data from the NEON trial may be made available following written request with appropriate scientific rationale and assurances, made to the trial steering group.

## Data-sharing statement

All data requests should be submitted to the corresponding author for consideration. Access to available anonymized data may be granted following review.

## Information governance statement

The University of Oxford is committed to handling all personal information in line with the UK Data Protection Act (2018) and the General Data Protection Regulation (EU GDPR) 2016/679. Under the Data Protection legislation, The University of Oxford is the Data Controller, and you can find out more about how we handle personal data, including how to exercise your individual rights and the contact details for our Data Protection Officer here: https://compliance.web.ox.ac.uk/individual-rights
