# Supplementary material for: The NEON (Nerve rEpair Or Not) trial: a randomized controlled trial of microsurgical repair versus nerve alignment for digital nerve injury
Source: Br J Surg. 2025 Sep 4;112(9):znaf174. doi: 10.1093/bjs/znaf174 (PMC12409716; doi:10.1093/bjs/znaf174)
Supplement: znaf174_Supplementary_Data [file znaf174_supplementary_data.zip › NEON_Protocol.pdf]

Study Title: A randomised controlled trial assessing if microsurgical nerve repair offers clinical benefit and cost effectiveness (in terms of patient–reported hand function, sensory recovery and adverse events) over exploration and washout without microsurgical nerve repair in adult patients with recent traumatic digital nerve injury.

Internal Reference Number / Short title: NEON – digital NErve, suture Or Not

Ethics Ref: 20/SC/0018

IRAS Project ID: 258872

Date and Version No: V3.0 02Jul2020

Chief Investigator: Professor Abhilash Jain, Associate Professor, University of Oxford

abhilash.jain@kennedy.ox.ac.uk

Investigators: Professor David Beard, University of Oxford

Associate Professor Jonathan Cook, University of Oxford

Mr Matthew Gardiner, University of Oxford

Dr Justin Wormald, University of Oxford

Mrs Cushla Cooper, University of Oxford

Professor Christina Jerosch-Herold, University of East Anglia

Mr Dominic Power, University Hospitals Birmingham NHS Foundation Trust

Dr Rafael Pinedo-Villanueva, University of Oxford

Sponsor: University of Oxford
Joint Research Office
1st floor, Boundary Brook House
Churchill Drive, Headington
Oxford, OX3 7GB

Funder: National Institute of Health Research, Health Technology Assessment

Chief Investigator Signature:

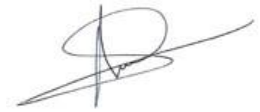

Statistician Signature:

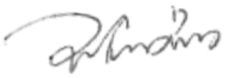

© Copyright: The University of Oxford and Oxford University Hospitals NHS Foundation Trust 2018

Please declare any potential conflicts of interest
Prof Christina Jerosch-Herold is a co-developer of the I-HaND questionnaire. It is freely available under a creative commons license for non-commercial research and Prof Jerosch-Herold does not derive any financial benefit from its use.

Confidentiality Statement
This document contains confidential information that must not be disclosed to anyone other than the Sponsor, the Investigator Team, Health Research Authority (HRA), host organisation, and members of the Research Ethics Committee (REC), unless authorised to do so.

© Copyright: The University of Oxford and Oxford University Hospitals NHS Foundation Trust 2018

TABLE OF CONTENTS

1. KEY CONTACTS 5
2. LAY SUMMARY 7
3. SYNOPSIS 8
4. ABBREVIATIONS 10
5. BACKGROUND AND RATIONALE 11
6. OBJECTIVES AND OUTCOME MEASURES 12
7. STUDY DESIGN 13
7.1. Pilot Phase 15
7.2. Study Participants 16
7.3. Inclusion Criteria 16
7.4. Exclusion Criteria 16
8. PROTOCOL PROCEDURES 17
8.1. Recruitment 17
8.2. Screening and Eligibility Assessment 18
8.3. Informed Consent 18
8.4. Randomisation 19
8.5. Blinding and code-breaking 19
8.6. Description of study intervention(s), comparators and study procedures (clinical) 20
8.7. Baseline Assessment 21
8.8. Operative Assessment 21
8.9. Subsequent Visits 21
8.9.1 Clinical appointments 21
8.9.2 Participant questionnaires 22
8.10. Long term follow up 23
8.11. Early Discontinuation/Withdrawal of Participants 23
8.12. Definition of End of Study 24
9. SAFETY REPORTING 24
9.1. Reporting Procedures for Unexpected Serious Events 24
9.2. Definition of Serious Adverse Events 25
10. STATISTICS AND ANALYSIS 25
10.1. Statistical Analysis Plan (SAP) 25
10.2. Description of the Statistical Methods 25

© Copyright: The University of Oxford and Oxford University Hospitals NHS Foundation Trust 2018

10.3. Sample Size Determination 25
10.4. Analysis populations 25
10.5. Decision points 26
10.6. The Level of Statistical Significance 26
10.7. Procedure for Accounting for Missing, Unused, and Spurious Data. 26
10.8. Health Economics Analysis 26
11. DATA MANAGEMENT 27
11.1. Source Data 27
11.2. Access to Data 27
11.3. Data Recording and Record Keeping 27
12. QUALITY ASSURANCE PROCEDURES 28
12.1. Risk assessment 28
12.2. Study monitoring 28
12.3. Study Committees 28
13. PROTOCOL DEVIATIONS 28
14. SERIOUS BREACHES 29
15. ETHICAL AND REGULATORY CONSIDERATIONS 29
15.1. Declaration of Helsinki 29
15.2. Guidelines for Good Clinical Practice 29
15.3. Approvals 29
15.4. Reporting 29
15.5. Participant Confidentiality 29
15.6. Expenses and Benefits 30
16. FINANCE AND INSURANCE 30
16.1. Funding 30
16.2. Insurance 30
16.3. Contractual arrangements 30
17. PUBLICATION POLICY 30
18. DEVELOPMENT OF A NEW PRODUCT/ PROCESS FOR THE GENERATION OF INTELLECTUAL PROPERTY (IP) 31
19. ARCHIVING 31
20. APPENDIX A: SCHEDULE OF STUDY PROCEDURES 31
21. APPENDIX B: AMENDMENT HISTORY 33
22. REFERENCES 33

© Copyright: The University of Oxford and Oxford University Hospitals NHS Foundation Trust 2018

1. KEY CONTACTS

**Chief Investigator**	**Prof Abhilash Jain**<br>Nuffield Department of Orthopaedics, Rheumatology and Musculoskeletal Sciences<br>Botnar Research Centre<br>University of Oxford<br>Nuffield Orthopaedic Centre<br>Windmill Road<br>Oxford<br>OX3 7LD<br>abhilash.jain@kennedy.ox.ac.uk
**Sponsor**	**University of Oxford**<br>Joint Research Office<br>First floor, Boundary Brook House<br>Churchill Drive, Headington<br>Oxford OX3 7GB<br>ctrg@admin.ox.ac.uk
**Funder(s)**	**National Institute of Health Research, Health Technology Assessment**<br>NIHR Evaluation, Trials and Studies Coordinating Centre<br>University of Southampton<br>Alpha House<br>Enterprise Road<br>Southampton<br>SO16 7NS<br>netsmonitoring@nihr.ac.uk<br>023 8059 5586
**Clinical Trials Unit**	**Surgical Intervention Trials Unit**<br>**Oxford Clinical Trials Research Unit**<br>**Trial Manager: Molly Glaze**<br>Nuffield Department of Orthopaedics, Rheumatology and Musculoskeletal Sciences<br>Botnar Research Centre<br>University of Oxford<br>Nuffield Orthopaedic Centre<br>Windmill Road<br>Oxford<br>OX3 7LD<br>neon@ndorms.ox.ac.uk<br>01865 223489

© Copyright: The University of Oxford and Oxford University Hospitals NHS Foundation Trust 2018

**Study statistician**	**Dr Ariel Wang**<br>Oxford Clinical Trials Research Unit<br>Centre for Statistics in Medicine<br>Nuffield Department of Orthopaedics, Rheumatology and Musculoskeletal Sciences<br>University of Oxford<br>Botnar Research Centre<br>Windmill Road<br>Oxford<br>OX3 7LD<br>ariel.wang@ndorms.ox.ac.uk
**Committees**	**Trial Steering Committee**<br>Chair: Professor Anne Schilder<br>UCL Ear Institute<br>Royal National Throat, Nose and Ear Hospital<br>330 Grays Inn Road<br>London WC1X 8DA<br>a.schilder@ucl.ac.uk<br><br>**Data Monitoring Committee**<br>Chair: Professor Chris Metcalfe<br>Canynge Hall<br>39 Whatley Road<br>Bristol BS8 2PS<br>Chris.metcalfe@bristol.ac.uk

© Copyright: The University of Oxford and Oxford University Hospitals NHS Foundation Trust 2018

2. LAY SUMMARY

Digital nerves are small nerves that pass along the side of each finger and provide sensation to the fingertips (see diagram). These nerves can be accidentally cut when handling sharp objects like a knife or broken glass. The NEON study aims to find out whether sewing the ends of the cut nerve surgically is beneficial or even needed. Thoroughly cleaning the cut wound before closing the skin is a much simpler procedure, and may be satisfactory for patients.

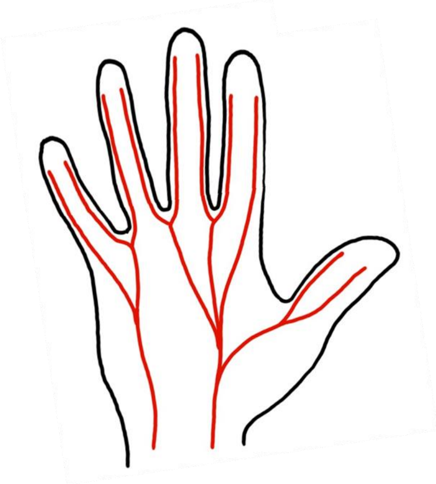

Diagram of right palm showing the digital nerves (in red)

There is some evidence that both treatments give good results. There is also some evidence that patients may not fully recover the feeling in their injured finger, even after sewing the nerve. Research so far has been conflicting and is of varying quality. For example, some studies do not directly compare treatments, or do not ask patients about their views of recovery.

The best way to find out if stitching the cut digital nerve is appropriate is to conduct a research study. NEON will compare surgical procedures for digital nerve injury, with or without stitches (also known as sutures). 478 patients will have one of these two treatment options by random allocation.

Patient questionnaires measuring fingertip sensation and quality of life will assess the benefit of each treatment up to 12 months after the operation. It will also be important to look into whether there is a difference in cost between the two treatments.

The study is supported by the specialist surgical societies in the UK such as the British Association of Plastic Surgery, the British Society for Surgery of the Hand and the Reconstructive Surgery Trials Network. Patient representatives who have had this injury are part of the study team and are members of the trial steering group.

The results of the study will be published via the funder's website and in medical journals. The study team will also present the results at academic conferences. The study website will present the results and a summary given to participants. To reach the patients and public more widely, the results will be made available through public websites like NHS Choices.

© Copyright: The University of Oxford and Oxford University Hospitals NHS Foundation Trust 2018

3. SYNOPSIS

Study Title	A randomised controlled trial to assess if microsurgical nerve repair offers clinical benefit and cost effectiveness (in terms of patient–reported hand function, sensory recovery and adverse events) over exploration and washout without microsurgical nerve repair in adult patients with recent traumatic digital nerve injury.		
Internal ref. no. / short title	**NEON** – Digital NErve, suture Or Not		
Study registration	ISRCTN: 16211574		
Sponsor	**University of Oxford**<br>Joint Research Office<br>1st floor, Boundary Brook House<br>Churchill Drive, Headington<br>Oxford, OX3 7GB		
Funder	**National Institute of Health Research, Health Technology Assessment**<br>NIHR Evaluation, Trials and Studies Coordinating Centre<br>University of Southampton<br>Alpha House<br>Enterprise Road<br>Southampton<br>SO16 7NS<br>netsmonitoring@nihr.ac.uk<br>023 8059 5586		
Study Design	Multi-centre, parallel, blinded (participant and assessor) two arm randomised controlled trial including economic analysis		
Study Participants	Patients 18 and over presenting with a single unilateral digital nerve injury appropriate for surgical repair.		
Sample Size	Randomised Trial: 478 participants, 239 in each treatment arm<br>Pilot phase focus groups: 4-8 participants in each group, 3-4 groups		
Planned Study Period	01 Oct 2019 – 30 Nov 2024<br>Total length of project: 62 months<br>Individual participant involvement: 12 months		
Planned Recruitment period	01 Apr 2020 – 31 Mar 2022<br>(embedded pilot phase 9 months from start of recruitment)		
Intervention(s)	Digital nerve surgery with microsurgical sutures.		
Comparator	Digital nerve surgery with realignment of nerve ends but no microsurgical sutures.		
	Objectives	Outcome Measures	Timepoint(s)
Primary	To ascertain the clinical effectiveness of microsurgical nerve repair for patients with digital nerve injuries.	Impact of Hand Nerve Disorders (I-HaND v2) PROM	6 weeks, 3 months and 12 months post randomisation

© Copyright: The University of Oxford and Oxford University Hospitals NHS Foundation Trust 2018

Secondary	To further assess neurosensory and functional recovery and health-related quality of life in the two surgical arms.	• Hand Health Profile of the Patient Evaluation Measure	12 months post randomisation
		• EQ-5D-5L index and –EQ-VAS	6 weeks, 3 months, 6 months and 12 months post randomisation
		• Static two-point discrimination test (2PD)<br>• Tactile gnosis using Shape/Texture Identification (STI) test<br>• Touch thresholds using Weinstein Enhanced Sensory Test (WEST) monofilaments	3 months and 12 months post randomisation (assessed at NEON clinical appointments)
	To examine the cost-effectiveness of microsurgical nerve repair.	• Health resource use questionnaires<br>• EQ-5D-5L index	6 weeks, 3 months, 6 months and 12 months post randomisation
	To compare complications of surgery and clinically problematic neuroma rates in the two surgical arms.	• Patient reported complication<br>• Clinical assessment (including Elliot score)<br>• Complications and further procedures in medical records	3 and 12 months post randomisation
		**Long term followup**<br>• Complications and further procedures based upon medical records and routine data	24 months post randomisation

© Copyright: The University of Oxford and Oxford University Hospitals NHS Foundation Trust 2018

4. ABBREVIATIONS

2PD	2 Point Discrimination
A&E	Accident & Emergency
BSSH	British Society for Surgery of the Hand
CI	Chief Investigator
CRF	Case Report Form
DMC	Data Monitoring Committee
EQ-5D-5L	Euroqol – 5 Dimensions – 5 Levels
GCP	Good Clinical Practice
ICER	Incremental Cost-Effectiveness Ratio
I-HaND	Impact of Hand Nerve Disorders
IP	Intellectual Property
HES	Hospital Episode Statistics
HRA	Health Research Authority
NICE	National Institute for Health and Care Excellence
OCTRU	Oxford Clinical Trials Research Unit
PROM	Patient Reported Outcome Measure
QALY	Quality Adjusted Life Year
RRAMP	Registration/Randomisation and Management of Product
REC	Research Ethics Committee
RSTN	Reconstructive Surgical Trainees Network
SAP	Statistical Analysis Plan
SD	Standard Deviation
SOP	Standard Operating Procedure
STI	Shape/Texture Identification
SWAT	Study Within A Trial
TMG	Trial Management Group
TSC	Trial Steering Committee
WEST	Weinstein Enhanced Sensory Test

© Copyright: The University of Oxford and Oxford University Hospitals NHS Foundation Trust 2018

5. BACKGROUND AND RATIONALE

Digital Nerve Injuries

Each finger has two digital nerves, one on its radial (lateral) and one on its ulnar (medial) palmar surface, supplying sensibility to the overlying skin on each side respectively. Injuries to these nerves are often sustained through sharp lacerations. Sensibility is lost when the nerves are injured. Digital nerve injuries can also be associated with injuries to the flexor tendons and/or finger joint capsules.

Based on an analysis of Hospital Episode Statistics (HES) data in England and Wales, approximately 3000 digital nerve repairs are undertaken in the UK annually (1, 2). These injuries account for 28,000 cases per year in Europe and 18,600 per year in the USA. They are by far the most common nerve injury treated surgically in the UK and represent 9% of all hand injuries (1, 2). The tariff for microsurgical repair of an isolated digital nerve injury in the UK is £2,610 according to the latest NHS Healthcare Resource Group code, which translates to a conservative estimate of the cost of microsurgical repair of digital nerve injuries in the UK at over £10 million a year (1, 2). This does not take into account patient related costs and loss to the economy due to time off work.

Current Practice

Treatment for digital nerve injuries involves referral to a specialist hand unit and direct end-to-end microsurgical repair of the cut nerve ends in an operating room by a specialist surgeon. The perceived advantage of performing surgical repair is that accurate coaptation of the nerve endings is more likely to result in a better outcome for patients and possibly fewer complications, such as neuroma formation.

Current Evidence

Clinical uncertainty surrounds the efficacy of digital nerve repair following traumatic digital nerve injury. A systematic review on the outcomes of repair in 2019 included all studies comparing documented surgical digital nerve repair with unrepaired injuries (3). This review highlighted the absence of randomised controlled trials amongst single centre consecutive case series (4). Results of non-comparative observational studies showed that surgical repair led to sensory recovery to pre-injury levels in a minority of patients (24%). Sensory function was determined in most cases by comparison of static 2 point discrimination (2PD) between the injured area and the contralateral, uninjured area. The most frequent primary outcomes assessed were not patient reported, and included assessment of hand function tending towards neurological clinical assessments of finger sensibility, assessed by spatial discrimination and detection threshold. Neither measures truly reflect the functional impact of sensory impairment due to patients adapting behaviour and assessor skill. Notably, in a study where the digital nerve was not repaired, the majority regained protective sensibility by 6 months and all patients declined further surgical intervention to improve their sensibility, suggesting satisfaction with the outcome (5).

A common justification by surgeons to undertake a digital nerve repair is to reduce the rate of nerve injury specific complications, such as neuroma, cold intolerance or other sensory disturbance. This was highlighted in a national survey (6). However, in contrast to the survey results, the systematic review indicates no evidence to support a hypothesis of higher adverse outcomes if a nerve is left unrepaired (neuroma rate 4.6% repaired versus 5% unrepaired) (5, 7-13).

The review also revealed deficiencies in clinician and patient communication. Some clinicians are likely not to be fully informing patients that their finger sensation may not return to pre-injury level, even with repair (14-19).

© Copyright: The University of Oxford and Oxford University Hospitals NHS Foundation Trust 2018

Evidence Based Surgery

The provision of surgical treatment of unknown efficacy or value is problematic for both individual patient wellbeing and the health service. Increased scrutiny by NHS England has led to four common hand surgical interventions being listed as "inappropriate procedures". As a result, the British Society for Surgery of the Hand (BSSH) has worked with NHS England and Clinical Commissioning Groups with the aim to provide evidence based recommendations for these conditions. Additionally, the BSSH has identified the repair of digital nerves as a health resource topic to study, confirmed through a James Lind Alliance Priority Setting Exercise (20).

Study Rationale

The limited work in this area highlights the uncertainty around whether there is a benefit to digital nerve repair. Responses from a survey of over 140 surgeons and therapists confirmed the existence of community equipoise. While some respondents stated that microsurgical repair is effective and essential, a considerable number were uncertain of efficacy, and confirmed the need for and willingness to engage with such a trial (6).

The NEON study seeks to answer whether microsurgical digital nerve repair is clinically effective, by comparing two surgical procedures. Given the lack of current evidence, the high frequency of the procedure and the lack of resources in the NHS, a robust trial is required to address the surgical dogma that surrounds this injury and its management.

Choice of Treatment Arms

The treatment arms for the study are detailed in section 8.6 and can be summarised as:

- Intervention: Digital nerve surgery with microsurgical sutures
- Comparator: Digital nerve surgery with realignment of nerve ends but no microsurgical sutures

Surgery with microsurgical sutures is the standard practice in the majority of NHS hospitals. Surgery without sutures was chosen as a comparator based on previous studies where the nerve was left unrepaired. Both groups will undergo treatment and some form of surgical operation. Hence the no suture group is not analogous to no treatment. The involvement of a surgical procedure in both groups protects against the known placebo effects of undergoing an operation. The design is therefore more akin to a comparison of two surgical interventions, identical except that one has the suspected critical surgical element omitted. Conducting both treatment arms in theatre allows specialist confirmation of diagnosis and participant blinding. It also allows those with concomitant injuries to have these repaired at the same time as their digital nerve surgery. During the study, the identification and referral of digital nerve injury from A&E will be evaluated (section 8.2). This will facilitate the possible extrapolation of study results into this setting.

6. OBJECTIVES AND OUTCOME MEASURES

Objectives	Outcome Measures	Timepoint(s) of evaluation of this outcome measure (if applicable)

© Copyright: The University of Oxford and Oxford University Hospitals NHS Foundation Trust 2018

**Primary Objective**<br>To ascertain the clinical effectiveness of microsurgical nerve repair for patients with digital nerve injuries.	Impact of Hand Nerve Disorders (I-HaND v2) PROM	6 weeks, 3 months and 12 months post randomisation
**Secondary Objectives**<br>1. To further assess neurosensory and functional recovery and health-related quality of life in the two surgical arms.	• Hand Health Profile of the Patient Evaluation Measure	12 months post randomisation
	• EQ-5D-5L and EQ-VAS	6 weeks, 3 months, 6 months and 12 months post randomisation
	• Static two-point discrimination test (2PD)<br>• Tactile gnosis using Shape/Texture Identification (STI) test<br>• Touch thresholds using Weinstein Enhanced Sensory Test (WEST) monofilaments	3 months and 12 months post randomisation (assessed at NEON clinical appointments)
2. To examine the cost-effectiveness of microsurgical nerve repair.	• Health resource use questionnaires<br>• EQ-5D-5L	6 weeks, 3 months, 6 months and 12 months post randomisation
3. To compare complications of surgery and clinically problematic neuroma rates in the two surgical arms.	• Patient reported complications<br>• Clinical assessment (including Elliot score)<br>• Complications and further procedures in medical records	3 and 12 months post randomisation
	**Long term followup**<br>• Complications and further procedures based upon medical records and routine data	24 months post randomisation

7. STUDY DESIGN

NEON is a multicentre randomised controlled trial with 1:1 allocation. Participants and follow-up assessors will be blinded to the randomised allocation.

Participants will be recruited to the study in hospitals across the UK. The study overall is modelled on 25 centres, but additional sites may be included depending on recruitment rates in the 9 month recruitment pilot phase.

Participants are expected to be enrolled in the study for up to 24 months. During the first 12 months the participants will attend follow-up clinic visits and complete questionnaires as per the schedule in Appendix

© Copyright: The University of Oxford and Oxford University Hospitals NHS Foundation Trust 2018

A and flow diagram (Figure 1). Routine data will be collected during the second 12 months via HES data and the Reconstructive Surgical Trainees Network (RSTN).

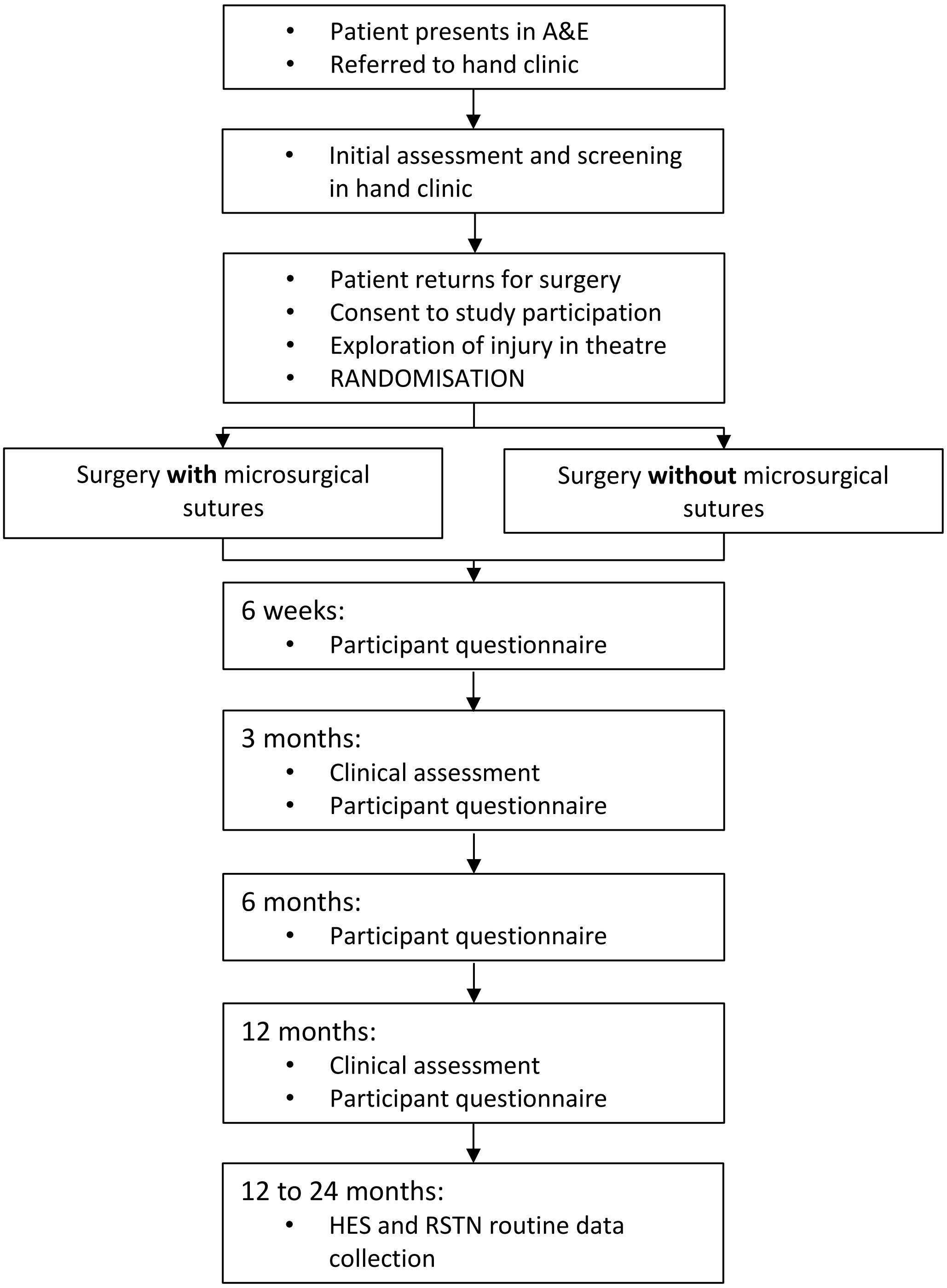

Figure 1: Participant flow diagram for NEON study

© Copyright: The University of Oxford and Oxford University Hospitals NHS Foundation Trust 2018

7.1. Pilot Phase

Incorporated within the NEON study design is a recruitment pilot phase, which will involve up to 9 centres with a staggered initiation over a period of 9 months. The aim of the pilot is to assess the following criteria: recruitment rate; ability to maintain assessor and participant blinding; adherence to the randomised procedure and early retention rates. The initial 9 centres will be used as a basis for revising aspects of study conduct.

Screening forms containing eligibility, reasons for exclusion and patients declining to participate will be completed. Rates of consent withdrawal and compliance with the randomised allocation will also be recorded. Any obvious barriers to recruitment will be assessed at 9 months. The overall recruitment target for the pilot is 44 participants, based on one participant per site per month with staggered site initiation.

The study team and oversight committees will consider all aspects of study conduct and any new evidence as the study moves into the definitive phase. Based on how the above criteria are met, the study team and committees will consider whether substantial changes are needed, and ultimately consider the viability of the study as currently designed and funded. Specific examples of this are seen in Table 1. Major changes derived from pilot work will be discussed with the appropriate stakeholders.

Table 1: *Success criteria within the pilot study*

	Red	Amber	Green
**Recruitment**			
**Sites open**<br>*target = 9*	2 (22%)	4 (44%)	7 (78%)
**Recruitment rate**<br>*target = 44, based on 1 per site per month, with staggered site opening*	10 (23%)	22 (50%)	33 (75%)
**Action**	Use other pilot data and patient focus group to inform discussion with TSC to consider stopping trial	Use other pilot data and patient focus group to inform discussion with TSC regarding strategies for improvement and consider changes to processes	Proceed to main trial – consider focus group feedback and other pilot data.
**Adherence**			
**Adherence to randomised procedure**	10 (23%)	22 (50%)	38 (85%)
**Action**	Consider screening log data and feedback from sites and discuss with TSC to consider stopping trial	Consider screening log data and feedback from sites and discuss with TSC strategies for improvement and consider changes to processes	Proceed to main trial – consider screening log data and feedback from sites.

© Copyright: The University of Oxford and Oxford University Hospitals NHS Foundation Trust 2018

Retention			
**PROMS collection and blinded assessment at 3 months** *(21 patients may reach 3 months follow up within the pilot)*	2 (10%)	7 (33%)	11 (52%)
**Action**	Use other pilot data and patient focus group to inform discussion with TSC to consider stopping trial	Use other pilot data and patient focus group to inform discussion with TSC regarding strategies for improvement and consider changes to processes	Proceed to main trial

Focus Groups

Participant focus groups will explore the study pathway and attitudes towards randomisation and blinding. The challenges of the study will be discussed, and the results will be used to further refine recruitment procedures, study information presentation and delivery, and training for clinical and research teams at sites.

Participants who agree to be contacted will be invited to take part in focus groups as part of the pilot phase. The focus groups will be held at the Botnar Research Centre in Oxford in the first instance. If participants from other sites express significant interest, efforts will be made to organise groups locally. All focus groups will be audio recorded and transcribed by a professional transcribing firm routinely sourced by the University of Oxford and versed in dealing with patient data. A confidentiality agreement will be put in place and any identifying information appearing in focus group transcripts will be removed as soon as possible following transcription to minimise risk of participant identification. Audio files will be treated as identifying data and will be excluded from archiving and sharing.

7.2. Study Participants

Patients 18 and over presenting with a single unilateral digital nerve injury appropriate for surgical repair will be considered for the NEON study.

7.3. Inclusion Criteria

Patients aged 18 years and above with a suspected complete digital nerve laceration in any single digit, including thumb and little finger, appropriate for surgical repair.

7.4. Exclusion Criteria

The participant may not enter the study if any of the following apply:

© Copyright: The University of Oxford and Oxford University Hospitals NHS Foundation Trust 2018

- Bilateral injury (ie both radial and ulnar digital nerves injured)
- Laceration outside the region between distal palmar crease and distal interphalangeal joint
- Closed injury
- Infected wounds
- Injuries in which a significant nerve gap exists which would preclude direct tension free surgical end-end repair
- Non-isolated or multi-level injury (ie common digital/wrist nerve injury, fracture)
- Unable to give consent
- Inability to comply with study follow-up procedures
- Date of surgery later than 10 days after injury
- Digital nerve incomplete or not present

Concomitant flexor tendon injuries and lacerations in 'critical zones' (Figure 2) will not be excluded. These will be minimisation factors in the randomisation, to balance the number of each in the two groups. Patients who are involved in current research can take part in NEON based on the clinical decision of the site team. The central study team can offer support with this decision.

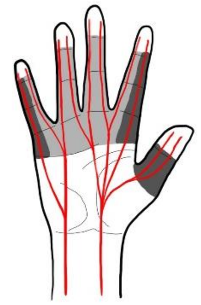

Figure 2. Schematic diagram showing digital nerve anatomy. Grey shading denotes zone of injury for inclusion into the trial. Dark shading denotes zone of injury in the 'critical zones', a minimisation factor in the study.

8. PROTOCOL PROCEDURES

8.1. Recruitment

The study will be undertaken across NHS hospitals in the UK. Potential participants will be recruited at specialist hand clinics following presentation at A&E or minor injury units. The clinical team will identify any potential participants and, if trained, discuss the study before referring on to the research team for further information. Potential participants will be able to discuss the study further with the local research team and, if agreeable, the patient will give informed consent. The research team at each site will comprise of clinical researchers and research nurses who will be trained in Good Clinical Practice (GCP) and Consent and be listed on the delegation log.

Patients will be given an information sheet and have the study explained to them by the researcher. They will be informed that participation in the study is optional and will not affect their medical or legal rights. All patients will be made aware of the study's aims, anticipated benefits and potential risks. An adequate amount of time will be given to allow them to make a decision.

Participants in the study will not be prioritised for surgery above those who are not taking part. They will proceed through the standard clinical pathway as per routine care. Participation in the study will only influence the type of procedure received.

© Copyright: The University of Oxford and Oxford University Hospitals NHS Foundation Trust 2018

8.2. Screening and Eligibility Assessment

Screening and eligibility assessment will take place pre-operatively, at outpatient clinics and on the day of surgery. The assessment will require examination of the finger with the dressings removed. Screening forms will be completed for each potential participant at each site, with reasons for ineligibility and non-participation documented.

The eligibility assessment is finalised in theatre via surgical exploration of the wound to confirm digital nerve injury immediately prior to randomisation.

Screening Study Within A Trial (SWAT)

The screening and identification of patients potentially requiring nerve repair can be problematic. Initial diagnosis of a digital nerve injury, which directs referral for further assessment and treatment in a specialist hand unit, is usually made by non-specialist personnel in an A&E department or minor injuries unit. This can generate over-referral to secondary care as referral to a hand unit is considered a safe option, despite the lack of need for surgery. Patient characteristics which generate excess referral often include symptoms of temporary numbness caused by neuropraxia or swelling, or presentation with a very distal injury which does not warrant repair.

This SWAT will explore the ability of referring personnel to accurately diagnose and detail digital nerve injuries in the A&E department by comparing pre-referral clinician findings to specialist clinician findings. Information on injury type and suspected structure damage will be collected from A&E during screening.

The intention of this SWAT is to provide information on the appropriateness of managing nerve injuries in A&E and to improve the current routine pathway. The results could lead to more accurate referrals to hand clinic for patients with digital nerve injuries, and facilitate training for the management of injuries that do not need referral.

8.3. Informed Consent

Informed consent will take place before surgery. The patient must personally sign and date the latest approved version of the Informed Consent Form before they can participate, and any study specific procedures are performed. Patients who consent but are found to have an injury that does not meet inclusion criteria during the operation will not proceed to be randomised. The informed consent process highlights this to patients.

Written and verbal versions of the Participant Information and Informed Consent will be presented to the participants detailing no less than: the exact nature of the study; what it will involve for the participant; the implications and constraints of the protocol; the known side effects and any risks involved in taking part. It will be clearly stated that the participant is free to withdraw from the study at any time for any reason without prejudice to future care, without affecting their legal rights, and with no obligation to give the reason for withdrawal.

The participant will be allowed as much time as wished to consider the information, and the opportunity to question the Investigator, their GP or other independent parties to decide whether they will participate in the study.

Written Informed Consent

Written Informed Consent will be obtained by means of a dated signature of the participant and of the person who presented and obtained the Informed Consent. The person who obtained the consent must

© Copyright: The University of Oxford and Oxford University Hospitals NHS Foundation Trust 2018

be suitably qualified and experienced and have been authorised to do so by the Chief/Principal Investigator. A copy of the signed Informed Consent will be given to the participant, and another copy will be kept in the medical notes. The original signed form will be retained in the study site file.

Patient Online Consent

Patients will be given the option to give their consent for the study online. If patients choose and after discussion with a research team member in clinic, a consent form will be emailed to patients for them to complete at home. Patients will have a copy of the Patient Information Sheet, and contact details for the local study teams and for the central team in Oxford are provided to allow the patient to ask any questions that they may have.

Patients click the link in their email to complete the form online. This process allows participants to consider the study in their own time and consent before they come to hospital for their surgery. Patients who do not complete this process before their day of operation but would still like to take part in the study can complete online in hospital if facilities are available, or can complete on paper.

8.4. Randomisation

Participants will be randomised in theatre after wound exploration and confirmation of digital nerve injury diagnosis. The randomisation will follow a 1:1 allocation ratio and use a minimisation algorithm; initially simple randomisation seeding will be used and minimisation will also incorporate a "random twist" element to protect allocation concealment throughout. During wound exploration, the surgeon will identify whether the injury involves flexor tendon damage or affects a "critical zone" (Figure 2). Randomisation will be minimised according to these two factors, as well as by study site.

Randomisation will be performed using a web based automated computer generated system. The allocation system will be generated by the trial statistician and will be programmed into the Oxford Clinical Trials Research Unit (OCTRU) computer randomisation system, Registration/Randomisation and Management of Product (RRAMP). The research team at each site will conduct the randomisation via secure log-ins to the web-based system, once informed consent has been given. An emergency randomisation list prepared by the trial statistician and held securely by the trial manager will be used if RRAMP is not available.

A member of the clinical research team delegated to treatment allocation (randomising) will enter the relevant data into the online randomisation system when the participant is in theatre, and inform the operating surgeon of the allocation details. If the participant is not under general anaesthetic, this will need to be done carefully without revealing the intended treatment to protect the blinding.

8.5. Blinding and code-breaking

Participants and follow-up assessors will be blinded to the randomised allocation. It is not possible to blind theatre staff.

Theatre equipment may provide visual and sound cues for participants under light or no sedation. The central study team will work with individual participating sites to agree a method of blinding that is practicable for them and meets the needs of the study.

Follow-up assessors will be blinded throughout the study to ensure objective sensory measurements. Participants in both arms will receive identical follow-up regimens to facilitate this. Site teams need to

© Copyright: The University of Oxford and Oxford University Hospitals NHS Foundation Trust 2018

ensure that those performing the blinded assessments are not in theatre and are not privy to unblinded discussions related to participants.

Unblinding

Those in the clinical team and the central study team will not be blinded to the allocation, so no code break procedure for clinical care or safety reporting is needed. The central study team will discuss specifically with each site which techniques they will employ to ensure participant and staff blinding. In any case in which unblinding is needed, or in which it occurs accidentally, the study team will follow processes as per the OCTRU Standard Operating Procedures (SOPs).

Perception of Blinding SWAT

Blinding participants and assessors to the study allocation is considered key to the interpretation of the study results. Knowledge of allocation can have a substantial effect on patient-reported outcomes and clinical assessments.

This SWAT aims to evaluate how well blinding is achieved, both for participants and assessors. Participants will be asked which treatment they think they received as part of their follow-up questionnaires. Assessors will be asked the same at the clinical assessments. Attitudes to blinding and to perception of treatment allocation will be explored with participants in the focus groups as part of the pilot phase.

The SWAT will demonstrate the reliability of participant and clinical blinding methods and the validity of the results within a context of blinding. It will facilitate the monitoring of blinding at sites, highlighting where efforts may need to be improved.

8.6. Description of study intervention(s), comparators and study procedures (clinical)

Surgical Preparation

The procedures will be undertaken as per NHS practice by specialist surgeons. They will be done either as a day case (vast majority) or as an inpatient, and will utilise either general or local anaesthesia as per clinical judgement.

Washout and exploration will be carried out in a sterile environment in an operating theatre as per standard practice. Exploration will include examination of the wound under anaesthesia with identification of all structures within the zone of injury, where applicable.

Surgery with microsurgical sutures

After exploration and washout, participants allocated to sutures will have this performed using end-to-end epineural sutures using, in the majority of cases, 8.0 or 9.0 synthetic non-absorbable suture under magnification (microscope or loupe).

Surgery without microsurgical sutures

After exploration and washout, participants allocated to no sutures will have the cut nerve ends realigned and laid back in-situ.

© Copyright: The University of Oxford and Oxford University Hospitals NHS Foundation Trust 2018

Completion of surgical procedure

Any associated injuries, such as flexor tendon injury, will be repaired, and the wound will be closed as per standard care. Dressings will be applied as per surgeon preference, and will depend on the extent of the injury, subsequent dissection and other associated injuries.

This intervention is a one-off treatment. Any further treatments will be sought as per routine NHS pathways.

Post-intervention rehabilitation

Simple (isolated) digital nerve injuries (without concomitant tendon or other tissue injury) are not routinely referred for hand therapy. For the purposes of this study, all participants with these simple injuries will be provided with a standardised advice sheet and a sensory relearning advice sheet, given out at discharge.

Participants with concomitant injuries will be referred to hand therapy for rehabilitation as per standard practice. Hand therapists will issue the same sensory relearning advice sheet when deemed safe for the participant to do the exercises without compromising tendon healing. Any hand therapy sessions will be logged at clinic visits.

8.7. Baseline Assessment

It is not possible to collect pre-injury baseline data. Injury details will be collected as part of the operative assessment, rather than as a separate baseline data.

8.8. Operative Assessment

At the time of surgery, the operating surgeon will confirm the diagnosis of a nerve laceration and the randomisation minimisation factors. Further details of the injury and procedure will be recorded. This will include adherence to the randomised allocation, the extent of concomitant injury repair needed and any complications.

8.9. Subsequent Visits

Follow-up will involve clinical appointments at 3 and 12 months post randomisation (supplementary to routine care), and participant questionnaires at 6 weeks, 3, 6 and 12 months post randomisation.

8.9.1 Clinical appointments

Current best evidence indicates that neurosensory recovery after this injury should be assessed using a variety of sensory tests, including touch threshold and spatial discrimination (21). These are recommended standardised tests for assessment of sensory function after nerve repair from the Swedish national quality registry for hand surgery (www.hakir.se) and the American Society of Hand Therapists Clinical Assessment Recommendations (22).

NEON clinical appointments are supplementary to standard care. They will be conducted by blinded research nurses or research therapists not involved in routine patient care. Blinded assessors will receive training on the standardised administration of measures through the provision of a manual and videos.

© Copyright: The University of Oxford and Oxford University Hospitals NHS Foundation Trust 2018

Further training workshops will be provided by the study team if deemed necessary following pilot review. The assessments should take approximately 30 minutes per participant and be conducted in a quiet environment, preferably an office or side room.

- Touch thresholds using Weinstein Enhanced Sensory Test (WEST) monofilaments.
  Assessors will follow a standardised protocol of randomised sequences to touch, or pretend to touch, the area impacted by the digital nerve injury 5 times with monofilaments. Doing this with different weighted filaments will allow the assessor to determine the lightest filament at which 4 out of 5 applications are correctly detected.

- Static two-point discrimination test (2PD) assessed with Dellon-Mackinnon DiskCriminator.
  The 2PD threshold will be determined by trials of 10 random applications of 1 or 2 points at different widths (between 2-30mm). The smallest distance at which participants identify 1 or 2 points correctly 7 out of 10 times will be recorded as the final threshold.

- Tactile gnosis using Shape/Texture Identification (STI) test
  Assessors will follow a standardised protocol, through which participants rely on tactile gnosis to identify three shapes (circle, square and hexagon) and three textures (sets of raised dots) fixed on three different discs. Participants work through 3 levels of smaller shapes and dots.

- Complications assessment
  Complications being reviewed include: neuroma, lack of sensitivity, hypersensitivity, cold intolerance, complex regional pain syndrome and flexor tendon re-rupture, stiffness and swelling.

8.9.2 Participant questionnaires

The following questionnaires will be sent to participants in the intervals above. These will be posted or emailed by the Clinical Trials Unit and can be completed by post or online. If participants do not respond within 2 weeks of sending the questionnaire, a reminder will be sent. Following this, if still no response, the central trial team may telephone the participant to collect the required data.

- Impact of Hand Nerve Disorder (I-HaND) v2
  I-HaND v2 is a validated patient-reported questionnaire specific to hand nerve disorder that measures symptoms, activity limitations and participation restrictions (23).

- Hand Health Profile of the Patient Evaluation Measure
  This questionnaire includes three sections: treatment, hand health profile and overall assessmsent. For the NEON study only section two, the hand health profile is being used. Similarly to I-HaND v2, this questionnaire asks about symptoms and activity limitations (24).

- EQ-5D-5L
  This questionnaire will be completed in order to calculate the EQ-5D-5L index (UK population weights) and EQ-VAS, and from this estimate quality adjusted life years for trial-based economic evaluation (25, 26).

- Complications
  Participants will report early complications 6 weeks post randomisation. These will include wound infection, delayed healing and re-opening, scar sensitivity and persistent ongoing pain.

© Copyright: The University of Oxford and Oxford University Hospitals NHS Foundation Trust 2018

- Health Resource Use

  Postoperative costs and lost productivity costs attributable to the injured digital nerve will be collected during the 12-month follow-up period. This includes information on outpatient visits, community care provision, and days off work due to injury.

8.10. Long term follow up

To obtain long term data about participants beyond the 12 month primary endpoint, data will be collected via two established methods:

Hospital Episode Statistics (HES) data

Routinely collected HES data will be used to determine re-operation rates on study participants 1-2 years following initial surgery. A bespoke HES extract linked to the Office of National Statistics data is required.

Long term follow up delivered by RSTN

On completion of 12 month follow up, long term data collection will take place using the RSTN. This will engage surgical trainees based at each of the study hospitals. They will review local routinely collected data of the study participants to identify any further procedures or complications (e.g. complex regional pain syndrome and neuroma formation). This data will be collected as part of a routine audit by RSTN.

8.11. Early Discontinuation/Withdrawal of Participants

During the course of the study, a participant may choose to withdraw from the randomised intervention or other study procedures at any time. This may happen for several reasons, including but not limited to:

- Investigator decision
  - Ineligibility (either arising during the study or retrospectively having been overlooked at screening)
  - Other clinical reasoning
  - Significant protocol deviation
  - Significant non-compliance with treatment regimen or study requirements
- Participant decision
  - The occurrence of what the participant perceives as an intolerable adverse event
  - Inability to comply with study procedures

The type of withdrawal and reason for withdrawal will be recorded in the relevant Case Report Form (CRF). If the participant is withdrawn due to an adverse event, the Investigator will arrange follow-up until the adverse event has resolved or stabilised.

Participants may decide any of the following types of withdrawal:

- Withdrawal from clinical assessment visits but willing to complete questionnaires at home.
- Withdrawal from clinical assessment visits and completing questionnaires, but willing for the study team to access medical records and any relevant hospital data that is recorded as part of routine care. These participants may also be willing to be followed up via HES and the RSTN.
- Withdrawal from all follow-up, but willing for data collected up to the point of withdrawal to be included in the final study analysis.

© Copyright: The University of Oxford and Oxford University Hospitals NHS Foundation Trust 2018

- Complete withdrawal, with data collected not being used in the final study analysis. (There are limits to this, for example when data has already been integrated into interim results.)

All participants who withdraw from the study will continue to receive routine clinical care.

Participants will not be replaced, as withdrawal and loss to follow-up has been accounted for in the estimated sample size. Analysis will be performed as per intention-to-treat, irrespective of compliance with treatment allocation.

8.12. Definition of End of Study

The end of the initial follow-up is defined as 30 days after the final participant visit, and after all the data has been entered and queries resolved. The end of the trial is defined as 1 year after this point, and when all longer term follow-up data has been entered, queries resolved, analysis completed and dissemination undertaken.

9. SAFETY REPORTING

The study involves no additional risks to participants beyond those of routine standard care. Participants will be informed of the standard risks associated with the anaesthetic and surgery.

Complications that the clinicians deem associated with the patient population and the study treatments will be reported by participants and assessed at study-specific clinic visits. Examples of expected complications include, but are not limited to:

- Wound infection, over granulation, delayed healing and re-opening
- Scar sensitivity, redness and abnormality
- Neuroma
- Lack of sensitivity
- Hypersensitivity
- Cold intolerance
- Complex regional pain syndrome
- Flexor tendon re-rupture, stiffness and swelling

Complications will be periodically reviewed by the Data Monitoring Committee (DMC) and any unusual increased patterns of serious adverse events (ie complications defined as serious) compared to what is expected for such patients and interventions will be notified to the REC.

9.1. Reporting Procedures for Unexpected Serious Events

An event occurring to a participant should be reported to the REC that gave a favourable opinion of the study where, in the opinion of the Chief Investigator (CI) the event was:

1. Serious according to the definitions in section 9.2
2. Related – resulted from administration of any of the research procedures
3. Unexpected in relation to those procedures

Sites need to submit reports of such events within 24 hours of the Principal Investigator becoming aware of the event. The central study team will then submit to the REC within 15 days of the CI becoming aware of the event.

© Copyright: The University of Oxford and Oxford University Hospitals NHS Foundation Trust 2018

No other adverse events will be reported.

9.2. Definition of Serious Adverse Events

A serious adverse event is any untoward medical occurrence that:

- results in death
- is life-threatening
- requires inpatient hospitalisation or prolongation of existing hospitalisation
- results in persistent or significant disability/incapacity
- consists of a congenital anomaly or birth defect.

Other ‘important medical events’ may also be considered a serious adverse event when, based upon appropriate medical judgement, the event may jeopardise the participant and may require medical or surgical intervention to prevent one of the outcomes listed above.

The term "life-threatening" in the definition of "serious" refers to an event in which the participant was at risk of death at the time of the event; it does not refer to an event which hypothetically might have caused death if it were more severe.

10. STATISTICS AND ANALYSIS

10.1. Statistical Analysis Plan (SAP)

The statistical aspects of the study are summarised here with details fully described in a statistical analysis plan that will be finalised before the final analysis takes place and agreed by the Trial Steering Committee (TSC).

10.2. Description of the Statistical Methods

The primary outcome measure (I-HaND v2) will be compared using a linear regression model with adjustment for the minimisation variables (critical or non-critical zone, associated tendon injury and study site). Study site will be accounted for using cluster robust variance. A secondary unadjusted analysis will also be carried out by an independent t-test. Secondary outcomes will be analysed using generalised linear models with adjustment for minimisation factors and baseline variables as appropriate. Where appropriate, repeated data will be used. Exploratory subgroup analyses will explore the possible treatment effect modification of clinically important factors (nerve injury type and critical location involvement or not), through the use of treatment by factor interaction, and will be interpreted cautiously.

10.3. Sample Size Determination

Based upon I-HaND data for patients with hand nerve conditions and our recent sample of digital nerve patients, the standard deviation (SD) could be as high as 21 points (23). To detect a target mean difference of 7 points (previous work in a similar measure suggests 7 would be an important difference; 0.3 standardised effect size) in the I-HaND with an SD of 21, 2-sided 5% significance level and 90% statistical power, 191 participants will be required per group (382 overall) (14, 27, 28). Based on the team’s experience of other digital nerve studies and hand trials 20% missing data has been allowed for giving an overall target of 478.

10.4. Analysis populations

© Copyright: The University of Oxford and Oxford University Hospitals NHS Foundation Trust 2018

All participants will be grouped according to their randomly allocated group (i.e. intention to treat/treatment policy) in the principal analyses. Complication data may also be presented (but not formally compared) by treatment received.

10.5. Decision points

No formal stopping rules are incorporated into the design and sample size, and accordingly no formal interim analyses are planned. The pilot phase will only assess trial feasibility, not clinical outcomes. An independent DMC will meet early in the course of the study to agree its terms of reference and will review confidential interim reports of accumulating data.

10.6. The Level of Statistical Significance

Statistical significance will be at the 5% level with corresponding confidence intervals derived whenever possible. Subgroup analyses will be at the same significance level but will be labelled “exploratory”.

10.7. Procedure for Accounting for Missing, Unused, and Spurious Data.

Principal analyses will be based upon observed data without imputation. The impact of missing data will also be explored in sensitivity analyses of the primary outcome and key secondary outcome (EQ-5D-5L) utilising using appropriate methods (e.g. the rctmiss Stata command for assessing the impact of missing not at random using a pattern mixed-model based approach) (29, 30).

10.8. Health Economics Analysis

An economic evaluation alongside the randomised controlled trial will be performed at 12-months post randomisation. The economic evaluation will be carried out on an intention-to-treat basis, from a NHS and personal and social services perspective including medical costs attributable to the digital nerve injury. Total costs and Quality Adjusted Life Years (QALYs) for both groups will be estimated. The mean differences in costs and QALYs between the two groups will be estimated using regression analyses controlling for baseline scores and trial stratification variables. The incremental cost-effectiveness ratio (ICER) will be calculated by dividing the difference in costs between the two treatment groups with the difference in QALYs.

The uncertainty surrounding the ICER will be estimated using non-parametric bootstrapping. To illustrate the statistical uncertainty surrounding the ICERs, the bootstrapped cost and QALYs pairs will be plotted on a cost-effectiveness (CE) plane (31). In a CE plane, the incremental costs between the two treatment groups will be plotted on the *y*-axis and the incremental QALYs on the *x*-axis. The cost-effectiveness acceptability curves (CEA curves) will be estimated to demonstrate the probability of cost-effectiveness of the two surgical procedures for a range of different willingness to pay thresholds. Following the recommendation from NICE, the treatment will be considered cost-effective when the ICER is less than £20,000 - £30,000 (32).

It is expected that the amount of missing data will be considerable, given the number and nature of healthcare resource use data and the different measurement points. Thus, multiple imputation methods (i.e. multiple imputation by chained equations) will be used for the main analysis to impute missing cost and QALYs data (33).

A number of secondary and sensitivity analyses will be undertaken to check the robustness of the results of the main analysis. A secondary analysis additional health outcomes will be considered, such as the I-

© Copyright: The University of Oxford and Oxford University Hospitals NHS Foundation Trust 2018

HaND. As a sensitivity analysis, the economic evaluation will be performed from a societal perspective including lost productivity costs, given the importance of returning to usual activities and work to the younger patients with a digital nerve injury. Another sensitivity analysis will be carried out including only complete cases, to explore the impact of multiple imputation on the results.

11. DATA MANAGEMENT

The data management aspects of the study are summarised here with details fully described in the Data Management Plan.

11.1. Source Data

Source documents are where data are first recorded, and from which participants' CRF data are obtained. These include, but are not limited to, hospital records (from which injury and hand therapy details may be summarised into the CRF), clinical and office charts, laboratory records, diaries, and correspondence.

CRF entries will be considered source data if the CRF is the site of the original recording (e.g. there is no other written or electronic record of data). All documents will be stored safely in confidential conditions. Data from participant questionnaires and from clinical assessments will be made available to sites to ensure the PI holds a contemporaneous copy. On all study-specific documents, other than the signed consent, the participant will be referred to by the study participant number/code, not by name.

11.2. Access to Data

Direct access will be granted to authorised representatives from the Sponsor and host institution for monitoring and/or audit of the study to ensure compliance with regulations.

On completion of the study, and with appropriate participant consent, fully anonymised data may be shared with other organisations at the behest of the funder. All requests for the use of the data from the NEON study will be approved by the CI, Trial Management Group (TMG) and where necessary the Trial Steering Committee (TSC). A data request form should be completed detailing the decision as to whether the request is accepted. In cases where individual site data is requested, only summary data would be provided with caveats for dissemination, to emphasise that study data should be interpreted as a whole.

11.3. Data Recording and Record Keeping

A Data Management and Sharing Plan will be produced for the study, which will describe the methods of data collection, entry and management, including details of data management tools and the study-specific database. All data will be processed in accordance with the Sponsor's policy for data protection.

All study-specific documents, except for the signed consent form and follow-up contact details, will refer to the participant with a unique study participant number/code and not by name. Participant identifiable data will be stored securely in accordance with OCTRU SOPs.

Site teams will enter data directly into the study-specific database, which will be validated and queried by central study team. The central team will control access to the database in accordance with OCTRU SOPs. Any paper questionnaires returned by participants will be stored securely in offices only accessible by swipe card by the central coordinating team staff in Oxford and authorised personnel.

© Copyright: The University of Oxford and Oxford University Hospitals NHS Foundation Trust 2018

Focus groups will be audio recorded in the field using a digital recording device; the resulting audio files (and accompanying field notes) will be transcribed for storage, and the original file deleted from the recording device, as soon as is practicably possible. Any identifying information appearing in focus group transcripts will be removed as soon as possible following transcription to minimise risk of participant identification. Transcription will be performed by a professional transcribing firm routinely sourced by the University of Oxford and versed in dealing with patient data. A confidentiality agreement will be put in place. Audio files will be treated as identifying data and will be excluded from archiving and sharing.

12. QUALITY ASSURANCE PROCEDURES

The study may be monitored or audited in accordance with the current approved protocol, GCP, relevant regulations and SOPs.

12.1. Risk assessment

OCTRU conducted a risk assessment prior to the study starting. Issues raised have been addressed within the final protocol and procedures have been planned to monitor the ongoing risks of the study. A risk proportionate approach will be utilised within this study. The risk assessment will be reviewed as necessary over the course of the study to reflect significant changes to the protocol or outcomes of monitoring activities.

12.2. Study monitoring

Regular central monitoring of study procedures will be imbedded into the study conduct and management, according to a study specific Monitoring Plan. The study will be subject to audit OCTRU Quality Assurance team, according to its Audit Programme. The study will also undergo a process of review before it is granted the green light to begin recruiting patients.

12.3. Study Committees

The Trial Management Group, Trial Steering Committee and Data Monitoring Committee will be set up and run in accordance to their Charters. All members have to sign to agree to the conditions of the Charter before sitting on a committee.

13. PROTOCOL DEVIATIONS

A study related deviation is a departure from:

- the ethically approved study protocol
- other study document or process (e.g. consent process or administration of study intervention)
- GCP
- any applicable regulatory requirements.

Any deviations from the protocol will be documented in a protocol deviation form and filed in the study master file. OCTRU SOPs will be followed for the procedure of identifying non-compliances, escalation to the central team and assessment of whether a non-compliance/deviation may be a potential serious breach.

© Copyright: The University of Oxford and Oxford University Hospitals NHS Foundation Trust 2018

14. SERIOUS BREACHES

A serious breach is a breach of the protocol or of the conditions or principles of GCP which is likely to affect to a significant degree –

(a) the safety or physical or mental integrity of the trial subjects; or

(b) the scientific value of the research.

In the event that a serious breach is suspected the Sponsor must be contacted within 1 working day. In collaboration with the CI, the serious breach will be reviewed by the Sponsor and, if appropriate, the Sponsor will report it to the approving REC and the relevant NHS host organisation within seven calendar days.

15. ETHICAL AND REGULATORY CONSIDERATIONS

Participants will be supported by their routine care providers over the duration of the study. The study intervention (digital nerve surgery) is a one-off procedure and all subsequent care will be sought through and provided by routine care.

15.1. Declaration of Helsinki

The Investigator will ensure that this study is conducted in accordance with the principles of the Declaration of Helsinki.

15.2. Guidelines for Good Clinical Practice

The Investigator will ensure that this study is conducted in accordance with relevant regulations and with GCP.

15.3. Approvals

Following Sponsor approval, the protocol and any patient-facing documentation will be submitted to an appropriate REC, HRA and host institutions for written approval. The Investigator will submit and, where necessary, obtain approval from the above parties for all amendments to the original approved documents.

15.4. Reporting

The CI shall submit once a year throughout the study, or on request, an Annual Progress report to the REC, HRA (where required) host organisation, Sponsor and funder (where required). In addition, an End of Study notification and final report will be submitted to the same parties.

15.5. Participant Confidentiality

The study will comply with the General Data Protection Regulation and Data Protection Act 2018, which require data to be de-identified as soon as it is practical to do so. The processing of the personal data of participants will be minimised by making use of a unique participant study number only on all study documents and any electronic database(s), with the exception of the CRF, where participant initials may

© Copyright: The University of Oxford and Oxford University Hospitals NHS Foundation Trust 2018

be added. All documents will be stored securely and only accessible by study staff and authorised personnel. The study staff will safeguard the privacy of participants' personal data.

15.6. Expenses and Benefits

Reasonable travel expenses for any visits additional to normal care will be reimbursed on production of receipts, or a mileage allowance provided as appropriate. Participants attending focus groups will receive a £20 shopping voucher to thank them for their time and participation, in addition to travel reimbursement as above.

16. FINANCE AND INSURANCE

16.1. Funding

The study is funded by the National Institute of Health Research, Health Technology Assessment (NIHR127807). The Nuffield Department of Orthopaedics, Rheumatology and Musculoskeletal Sciences at the University of Oxford will manage the finances and budget.

16.2. Insurance

The University has a specialist insurance policy in place which would operate in the event of any participant suffering harm as a result of their involvement in the research (Newline Underwriting Management Ltd, at Lloyd's of London). NHS indemnity operates in respect of the clinical treatment that is provided.

16.3. Contractual arrangements

Appropriate contractual arrangements will be put in place with all third parties.

17. PUBLICATION POLICY

This study is funded by the NIHR Health Technology Assessment Programme and carried out in collaboration with the RSTN. Any publication arising out of the study will follow the NIHR publication policy. The Investigators will be involved in reviewing drafts of the manuscripts, abstracts, press releases and any other publications arising from the study. Authorship will be determined in accordance with the International Committee of Medical Journal Editors guidelines and other contributors will be acknowledged.

All investigators and co-ordinators who take part in the study will be designated as members of the "NEON Trial Group" and will be publicly listed on the study website. All NEON publications will be published on behalf of the "NEON Trial Group", which means all trial group members can list these in their curriculum vitae. All members of the "NEON Trial Group" will be submitted to be listed and citable in PubMed.

Study results will be published via the funder's website and in medical journals. The study team will present the results at academic conferences. The study website will present the results and a summary given to participants. Making the results available through public websites like NHS Choices will allow them to reach patients and the public more widely.

© Copyright: The University of Oxford and Oxford University Hospitals NHS Foundation Trust 2018

18. DEVELOPMENT OF A NEW PRODUCT/ PROCESS FOR THE GENERATION OF INTELLECTUAL PROPERTY (IP)

Ownership of IP generated by employees of the University vests in the University. The protection and exploitation of any new IP is managed by the University's technology transfer office, Oxford University Innovations.

19. ARCHIVING

The Trial Master File including all essential documents must be retained for at least 5 years after the completion of study-related activities. The Sponsor File will be archived centrally, and the Investigator Site Files will be archived at site.

20. APPENDIX A: SCHEDULE OF STUDY PROCEDURES

NB only inclusive of the first 12 months of study participation

**Procedures**	**Visits**					
	**Screening**	**Operation**	**6 weeks**	**3 months**	**6 months**	**12 months**
Eligibility assessment	X	X				
Informed consent		X				
Demographics		X				
Injury details		X				
Randomisation		X				
Surgery details		X				
Impact of Hand Nerve Disorders (I-HaND)			X	X		X
Hand Health Profile of the Patient Evaluation Measure						X
EQ-5D-5L questionnaire			X	X	X	X
Health resource use questionnaires				X	X	X
Perception of treatment						X
Static two-point discrimination test				X		X
Tactile gnosis using Shape/Texture Identification test				X		X
Touch thresholds using Weinstein Enhanced Sensory Test monofilaments				X		X

© Copyright: The University of Oxford and Oxford University Hospitals NHS Foundation Trust 2018

Participant-reported complications			X			
Complications review				X		X
Elliot score				X		X
Hand rehabilitation log				X		
Adverse event assessments		X		X		X

© Copyright: The University of Oxford and Oxford University Hospitals NHS Foundation Trust 2018

21. APPENDIX B: AMENDMENT HISTORY

All amendments will only be implemented after the necessary approvals have been obtained and after all relevant parties have been notified of the changes.

Amendment No.	Protocol Version No.	Date issued	Author(s) of changes	Details of Changes made
Minor amendment 2	V2.0	04Feb2020	Molly Glaze<br>Vicki Barber<br>Beverly Shirkey<br>Jonathan Cook	- Clarification on outcomes in section 3 and 6<br>- Admin changes for trial/study and patient/participant<br>- Staff changes
Substantial amendment 3	V3.0	02Jul2020	Molly Glaze<br>Ariel Wang	- Addition of RAG table<br>- Correction of online consent process<br>- Addition of all outcome timepoints in summary tables<br>- Clarification of EQ-5D as an outcome measure<br>- Staff update: statistician change from Beverly Shirkey to Ariel Wang

22. REFERENCES

1. Manley OWG, Wormald JCR, Furniss D. The changing shape of hand trauma: an analysis of Hospital Episode Statistics in England. J Hand Surg Eur Vol. 2019;44(5):532-6.
2. Centre. HaSCI. Hospital Episode Statistics.
3. Dunlop RLE, Wormald JCR, Jain A. Outcome of surgical repair of adult digital nerve injury: a systematic review. BMJ Open. 2019;9(3):e025443.
4. Lundborg G, Rosen B. Hand function after nerve repair. Acta Physiol (Oxf). 2007;189(2):207-17.
5. Chow SP, Ng C. Can a divided digital nerve on one side of the finger be left unrepaired? Journal of Hand Surgery. 1993;18 B(5):629-30.
6. Wormald JCR, Gardiner MD, Jain A. To repair or not repair a single digital nerve in adults? J Hand Surg Eur Vol. 2019;44(6):655-6.
7. Sullivan DJ. Results of digital neurorrhaphy in adults. Journal of hand surgery (Edinburgh, Lothian). 1985;10(1):41-4.
8. Shaffer JM, Cleveland F. Delayed Suture of Sensory Nerves of the Hand. Annals of surgery. 1950;131(4):556-63.
9. Buncke Jr HJ. Digital nerve repairs. Surgical Clinics of North America. 1972;52(5):1267-85.
10. Pereira JH, Bowden REM, Gattuso JM, Norris RW. Comparison of results of repair of digital nerves by denatured muscle grafts and end-to-end sutures. Journal of Hand Surgery. 1991;16 B(5):519-23.
11. Tadjalli HE, McIntyre FH, Dolynchuk KN, Murray KA. Digital nerve repair: Relationship between severity of injury and sensibility recovery. Annals of Plastic Surgery. 1995;35(1):36-40.
12. Fakin RM, Calcagni M, Klein HJ, Giovanoli P. Long-term clinical outcome after epineural coaptation of digital nerves. The Journal of hand surgery, European volume. 2016;41(2):148-54.
13. Khuc T, Leclercq D, Carlier A. Microsurgical repair of 110 digital nerves. Acta Chirurgica Belgica. 1982;82(3):271-80.

© Copyright: The University of Oxford and Oxford University Hospitals NHS Foundation Trust 2018

14. Al-Ghazal SK, McKiernan M, Khan K, McCann J. Results of clinical assessment after primary digital nerve repair. Journal of Hand Surgery. 1994;19 B(2):255-7.
15. Altissimi M, Mancini GB, Azzara A. Results of primary repair of digital nerves. Journal of Hand Surgery. 1991;16 B(5):546-7.
16. Chaise F, Friol JP, Gaisne E. Results of emergency repair of wounds of palmar collateral nerves of the fingers. Revue de chirurgie orthopedique et reparatrice de l'appareil moteur. 1993;79(5):393-7.
17. Cheng AS, Hung L, Wong JM, Lau H, Chan J. A prospective study of early tactile stimulation after digital nerve repair. Clinical orthopaedics and related research. 2001(384):169-75.
18. Hohendorff B, Staub L, Fritsche E, von Wartburg U. Sensory nerve function after unilateral digital vascular-nerve injury: nerve repair with and without arterial repair. Handchirurgie, Mikrochirurgie, plastische Chirurgie : Organ der Deutschsprachigen Arbeitsgemeinschaft fur Handchirurgie : Organ der Deutschsprachigen Arbeitsgemeinschaft fur Mikrochirurgie der Peripheren Nerven und Gefasse : Organ der Vereinigung der Deutschen Plastischen Chirurgen. 2009;41(5):306-11.
19. Lohmeyer JA, Hulsemann W, Mann M, Schmauss D, Machens HG, Habenicht R. Return of sensitivity after digital nerve reconstruction in children: How does age affect outcome? Handchirurgie Mikrochirurgie Plastische Chirurgie. 2013;45(5):265-70.
20. James Lind Alliance and BSSH Priority Setting Partnership of the Top 10 Research Priorities for the Hand and Wrist 2017 [Available from: http://www.bssh.ac.uk/patients/bssh_james_lind_alliance_partnership.aspx. [
21. Miller LK, Chester R, Jerosch-Herold C. Effects of Sensory Reeducation Programs on Functional Hand Sensibility after Median and Ulnar Repair: A Systematic Review. Journal of Hand Therapy. 2012;25(3):297-307.
22. Jerosch-Herold C. Chapter 3: Sensibility. In: MacDermid JC, editor. ASHT Clinical Assessment Recommendations. 3rd ed. New Jersey: American Society of Hand Therapists; 2015.
23. Ashwood M, Jerosch-Herold C, Shepstone L. Development and validation of a new patient-reported outcome measure for peripheral nerve disorders of the hand, the I-HaND(c) Scale. J Hand Surg Eur Vol. 2018;43(8):864-74.
24. Dias JJ, Bhowal B, Wildin CJ, Thompson JR. Assessing the outcome of disorders of the hand. Is the patient evaluation measure reliable, valid, responsive and without bias? J Bone Joint Surg Br. 2001;83(2):235-40.
25. Herdman M, Gudex C, Lloyd A, Janssen M, Kind P, Parkin D, et al. Development and preliminary testing of the new five-level version of EQ-5D (EQ-5D-5L). Qual Life Res. 2011;20(10):1727-36.
26. Janssen MF, Pickard AS, Golicki D, Gudex C, Niewada M, Scalone L, et al. Measurement properties of the EQ-5D-5L compared to the EQ-5D-3L across eight patient groups: a multi-country study. Qual Life Res. 2013;22(7):1717-27.
27. Cook JA, Julious SA, Sones W, Hampson LV, Hewitt C, Berlin JA, et al. DELTA² guidance on choosing the target difference and undertaking and reporting the sample size calculation for a randomised controlled trial. BMJ. 2018;363:k3750.
28. Cook JA, Hislop J, Adewuyi TE, Harrild K, Altman DG, Ramsay CR, et al. Assessing methods to specify the target difference for a randomised controlled trial: DELTA (Difference ELicitation in TriAls) review. Health Technol Assess. 2014;18(28):v-vi, 1-175.
29. White IR, Kalaitzaki E, Thompson SG. Allowing for missing outcome data and incomplete uptake of randomised interventions, with application to an Internet-based alcohol trial. Stat Med. 2011;30(27):3192-207.
30. White IR, Horton NJ, Carpenter J, Pocock SJ. Strategy for intention to treat analysis in randomised trials with missing outcome data. BMJ. 2011;342:d40.
31. Black WC. The CE plane: a graphic representation of cost-effectiveness. Med Decis Making. 1990;10(3):212-4.
32. McCabe C, Claxton K, Culyer AJ. The NICE cost-effectiveness threshold: what it is and what that means. Pharmacoeconomics. 2008;26(9):733-44.
33. Azur MJ, Stuart EA, Frangakis C, Leaf PJ. Multiple imputation by chained equations: what is it and how does it work? Int J Methods Psychiatr Res. 2011;20(1):40-9.

© Copyright: The University of Oxford and Oxford University Hospitals NHS Foundation Trust 2018

© Copyright: The University of Oxford and Oxford University Hospitals NHS Foundation Trust 2018
