# Supplementary material for: The NEON (Nerve rEpair Or Not) trial: a randomized controlled trial of microsurgical repair versus nerve alignment for digital nerve injury
Source: Br J Surg. 2025 Sep 4;112(9):znaf174. doi: 10.1093/bjs/znaf174 (PMC12409716; doi:10.1093/bjs/znaf174)
Supplement: znaf174_Supplementary_Data [file znaf174_supplementary_data.zip › Supplementary_Material.docx]

The NEON (Nerve rEpair Or Not) Trial: a randomised controlled trial of microsurgical repair versus nerve alignment for digital nerve injury

Justin C R Wormald^1,2±^, Matthew D. Gardiner^1,3±^ , Christina Jerosch-Herold^4^, Jonathan Cook^1^, Rafael Pinedo Villanueva^1^, Ciaron O’Hanlon^1^, Naomi Vides^1^, Gianluca Fabiano^1^, Scott Parsons^1^, Loretta Davies^1^, Heidi Fletcher^1^, Molly Glaze^1^, Cushla Cooper^1^, Dominic Power^5^, Abhilash Jain*^1,6^, David Beard*^1,7^ on behalf of the NEON study team

1. Nuffield Department of Orthopaedics, Rheumatology and Musculoskeletal Sciences, University of Oxford, Oxford, UK
2. Department of Plastic and Reconstructive Surgery, Oxford University Healthcare NHS Foundation Trust, Oxford, UK
3. Department of Plastic and Reconstructive Surgery, Frimley Health NHS Foundation Trust, Slough, UK
4. Rehabilitation Research, School of Health Sciences, University of East Anglia, Norwich, UK
5. Birmingham Hand Centre, Queen Elizabeth Hospital, Birmingham, UK
6. Department of Plastic and Reconstructive Surgery, Imperial College Healthcare NHS Trust, London, UK
7. NHMRC Clinical Trials Centre, Faculty of Medicine & Health, University of Sydney, Australia

**Corresponding author:**

Justin Wormald

[justin.wormald@ndorms.ox.ac.uk](mailto:justin.wormald@ndorms.ox.ac.uk)

Surgical Interventional Trials Unit (SITU), Nuffield Department of Orthopaedics, Rheumatology and Musculoskeletal Sciences, University of Oxford, Oxford, UK

ORCID: 0000-0001-6197-4094

X: @JCRWormald

**Supplementary Materials - Index**

| **Supplementary Tables** | *Page* |
| --- | --- |
| ***Supplementary Table 1:*** *Randomisation factors at baseline – split by treatment group and overall* | *4* |
| ***Supplementary Table 2:*** *Details of interventions received split by randomised treatment group* | *5* |
| ***Supplementary Table 3:*** *Details of surgery summarised by treatment group* | *5* |
| ***Supplementary Table 4:*** *Details of surgery – Suture repair arm only* | *7* |
| ***Supplementary Table 5:*** *Complications experienced during surgery* | *8* |
| ***Supplementary Table 6:*** *Completion of follow-up questionnaires* | *8* |
| ***Supplementary Table 7:*** *Withdrawals from follow-up split by treatment group* | *9* |
| ***Supplementary Table 8:*** *Missing data in I-HaND (v2) scores by treatment group and timepoint* | *10* |
| ***Supplementary Table 9****: Results of the hand health profile by treatment groups – ITT population* | *10* |
| ***Supplementary Table 10:*** *Results of the EQ-5D-5L index and the EQ-VAS by treatment groups – ITT population* | *10* |
| ***Supplementary Table 11:*** *Results of Patient’s Global Rating of Sensation by treatment groups – ITT population* | *11* |
| ***Supplementary Table 12:*** *Results of Touch thresholds using Weinstein Enhanced Sensory Test (WEST) by treatment groups (3 levels)* | *12* |
| ***Supplementary Table 13:*** *Results of the Static two-point discrimination test (2PD) by treatment groups – ITT population* | *12* |
| ***Supplementary Table 14:*** *Results of Tactile gnosis using Shape/Texture Identification test (STI) by treatment groups – ITT population* | *13* |
| ***Supplementary Table 15:*** *Complications experienced and reviewed by health professional at 6 weeks post randomisation* | *13* |
| ***Supplementary Table 16:*** *Complications and further procedures in medical records at 3 months and 12 months post randomisation* | *13* |
| ***Supplementary Table 17:*** *Summary of cold intolerance at 3 months and 12 months post randomisation by treatment groups – ITT population* | *14* |
| ***Supplementary Table 18:*** *Analysis of cold intolerance at 3 months and 12 months post randomisation by treatment groups – ITT population* | *15* |
| ***Supplementary Table 19:*** *Summary of Elliot score items at 3 months and 12 months post randomisation by treatment groups – ITT population* | *15* |
| ***Supplementary Table 20:*** *Analysis of Elliot score at 3 months and 12 months post randomisation by treatment groups – ITT population* | *16* |
| ***Supplementary Table 21:*** *Details of serious adverse events* | *16* |
| **Supplementary Information** |  |
| *NEON trial team details* | *17* |

**Supplementary Tables**

***Supplementary Table 1:*** *Randomisation factors at baseline – split by treatment group and overall*

|  | **Suture repair  (n=61)** | **Nerve alignment alone  (n=61)** | **Total  (n=122)** |
| --- | --- | --- | --- |
| **Site^1^** | | | |
| Frimley Health NHS Foundation Trust | 2 (3.28) | 2 (3.28) | 4 (3.28) |
| Hampshire Hospitals NHS Foundation Trust | 2 (3.28) | 2 (3.28) | 4 (3.28) |
| Imperial College Healthcare NHS Trust | 2 (3.28) | 5 (8.20) | 7 (5.74) |
| Mid and South Essex NHS Foundation Trust | 2 (3.28) | 3 (4.92) | 5 (4.10) |
| Norfolk and Norwich University Hospitals NHS Foundation Trust | 1 (1.64) | 0 (0.00) | 1 (0.82) |
| Oxford University Hospitals NHS Foundation Trust | 12 (19.67) | 10 (16.39) | 22 (18.03) |
| Queen Victoria Hospital NHS Foundation Trust | 3 (4.92) | 4 (6.56) | 7 (5.74) |
| Royal Cornwall Hospitals NHS Trust | 7 (11.48) | 7 (11.48) | 14 (11.48) |
| Royal Devon University Healthcare NHS Foundation Trust | 0 (0.00) | 1 (1.64) | 1 (0.82) |
| South Tees Hospitals NHS Foundation Trust | 2 (3.28) | 0 (0.00) | 2 (1.64) |
| Stoke Mandeville Hospital | 13 (21.31) | 12 (19.67) | 25 (20.49) |
| The Newcastle upon Tyne Hospitals NHS Foundation Trust | 1 (1.64) | 1 (1.64) | 2 (1.64) |
| University Hospitals Coventry and Warwickshire NHS Trust | 3 (4.92) | 2 (3.28) | 5 (4.10) |
| University Hospitals of Derby and Burton NHS Foundation Trust | 11 (18.03) | 12 (19.67) | 23 (18.85) |
| **Flexor Tendon Damage^1^** | | | |
| Yes | 16 (26.23) | 16 (26.23) | 32 (26.23) |
| No | 45 (73.77) | 45 (73.77) | 90 (73.77) |
| **In Critical Zone^1^** | | | |
| Yes | 45 (73.77) | 45 (73.77) | 90 (73.77) |
| No | 16 (26.23) | 16 (26.23) | 32 (26.23) |
| ^1^Summaries are n (%) | | | |

***Supplementary Table 2:*** *Details of interventions received split by randomised treatment group*

|  | **Suture repair  (n=61)** | **Nerve alignment alone  (n=61)** |
| --- | --- | --- |
| Received allocated intervention as planned^12^ | 61 (100.00) | 57 (93.44) |
| Didn’t receive allocated intervention as planned^2^ | 0 (0.00) | 4 (6.56) |
| Received alternate trial treatment^2^ | 0 (0.00) | 4 (6.56) |
| Surgeon opted to repair^2^ | 0 (0.00) | 4 (6.56) |
| ^1^Participants are classed as having received their allocated intervention if for those randomised to surgical exploration and suture repair had microsurgical sutures placed as planned, and those who were randomised to surgical exploration with nerve alignment did not undergo suture repair. | | |
| ^2^Summaries are n (%). | | |

***Supplementary Table 3:*** *Details of surgery summarised by treatment group*

|  | **Suture repair  (n=61)** | **Nerve alignment alone  (n=61)** | **Total  (n=122)** |
| --- | --- | --- | --- |
| **Time from randomisation to surgery (days)^13^** | 0.03 (0.26), 0.00 [0.00, 0.00],  {0.00, 2.00} | 0.00 (0.00), 0.00 [0.00, 0.00],  {0.00, 0.00} | 0.02 (0.18), 0.00 [0.00, 0.00],   {0.00, 2.00} |
| **Time in theatre (minutes)^1^** | 80.93 (33.88), 72.50 [60.00, 97.75],  {26.00, 175.00} | 67.70 (35.27), 60.00 [50.00, 86.00],  {5.00, 157.00} | 74.15 (35.09), 66.00 [51.50, 90.00],   {5.00, 175.00} |
| **Grades of surgeons^2^** | | | |
| Consultant | 19 (31.15) | 13 (21.31) | 32 (26.23) |
| SpR | 23 (37.70) | 34 (55.74) | 57 (46.72) |
| CT | 12 (19.67) | 8 (13.11) | 20 (16.39) |
| Other | 7 (11.48) | 6 (9.84) | 13 (10.66) |
| **Type of anaesthetic^2^** | | | |
| General | 5 (8.20) | 7 (11.48) | 12 (9.84) |
| Local | 55 (90.16) | 51 (83.61) | 106 (86.89) |
| Both | 1 (1.64) | 3 (4.92) | 4 (3.28) |
| **Blinding of the patient^2^** | | | |
| Finger hidden | 55 (100.00) | 49 (96.08) | 104 (85.25) |
| Treatment allocation communication hidden | 54 (98.18) | 48 (94.12) | 102 (83.61) |
| Other blinding measure^4^ | 10 (19.61) | 5 (11.36) | 15 (12.30) |
| **Digital artery divided^2^** | | | |
| Yes | 33 (55.00) | 35 (59.32) | 68 (55.74) |
| No | 27 (45.00) | 24 (40.68) | 51 (41.80) |
| **Digital artery repaired^2^** | | | |
| Yes | 8 (13.11) | 5 (8.47) | 13 (10.66) |
| No | 53 (86.89) | 54 (91.53) | 107 (87.70) |
| **Flexor tendon injured^2^** | | | |
| FDS radial slip | 5 (8.20) | 3 (4.92) | 8 (6.56) |
| FDS ulnar slip | 3 (4.92) | 4 (6.56) | 7 (5.74) |
| FDS main tendon | 0 (0.00) | 2 (3.28) | 2 (1.64) |
| FDP | 12 (19.67) | 13 (21.31) | 25 (20.49) |
| **Flexor tendon reparied^2^** | | | |
| FDS radial slip | 4 (6.56) | 3 (4.92) | 7 (5.74) |
| FDS ulnar slip | 2 (3.28) | 0 (0.00) | 2 (1.64) |
| FDS main repair | 8 (13.11) | 11 (18.03) | 19 (15.57) |
| FDP | 8 (13.11) | 11 (18.03) | 19 (15.57) |
| **Hospital stay^2^** | | | |
| Day case | 58 (95.08) | 59 (96.72) | 117 (95.90) |
| Inpatient stay | 3 (4.92) | 2 (3.28) | 5 (4.10) |
| ^1^Summaries are Mean (SD), Median[IQR], {Range} | | | |
| ^2^Summaries are n (%) | | | |
| ^3^Two patient had dates of surgery recorded before date of randomisation. These were data errors and were updated to have date or surgery equal to date of randomisation. | | | |
| ^4^See Appendix for table with other blinding measures | | | |

***Supplementary Table 4:*** *Details of surgery – Suture repair arm only*

|  | **Suture repair  (n=61)** | **Nerve alignment alone  (n=4)^3^** |
| --- | --- | --- |
| **Number of sutures used^1^** | 3.09 (1.12) 3.00 (2.75, 4.00) (1.00, 6.00) | 3.25 (1.71) 3.50 (2.50, 4.25) (1.00, 5.00) |
| **Type of sutures^2^** | | |
| 8.0 non-absorbable | 23 (37.70) | 3 (75.00) |
| 9.0 non-absorbable | 38 (62.30) | 1 (25.00) |
| Other | 0 (0.00) | 0 (0.00) |
| **Loupe magnification used^2^** | | |
| Yes | 45 (76.27) | 2 (66.67) |
| No | 14 (23.73) | 1 (33.33) |
| **End-to-end alignment with gapping less than 1mm^2^** | | |
| Yes | 54 (91.53) | 3 (100.00) |
| No | 5 (8.47) | 0 (0.00) |
| **Fascicle extrusion^2^** | | |
| Yes | 5 (8.47) | 0 (0.00) |
| No | 54 (91.53) | 3 (100.00) |
| ^1^Summaries are Mean(SD), Median[IQR], {Range} | | |
| ^2^Summaries are n (%) | | |
| ^3^Note, 4 participants in nerve alignment (Nerve alignment alone) group received Suture repair (non-compliance), hence being included in this table. | | |

***Supplementary Table 5:*** *Complications experienced during surgery*

|  | **Suture repair** | **Nerve alignment alone** |
| --- | --- | --- |
| **Participants with complications during surgery** | 2 | 1 |
| Anaesthetic related complications | 0 | 0 |
| Procedure abandoned | 0 | 0 |
| Equipment issues | 0 | 0 |
| Other, specified as follows | 2 | 1 |
| delay due to difficulty accessing redcap and randomising | 1 | 0 |
| Patient could not tolerate tourniquet for length of operation | 0 | 1 |
| Supraventricular Tachycardia | 1 | 0 |

***Supplementary Table 6:*** *Completion of follow-up questionnaires*

|  | **Received (%)** |
| --- | --- |
| **Randomisation theatre screening^1^** | 122 (100) |
| **Post randomisation operative details^1^** | 122 (100) |
| **6 weeks questionnaire^1^** |  |
| I-HaND | 81 (66.4) |
| EQ-5D-5L | 77 (63.1) |
| Patient reported complications | 82 (67.2) |
| **3 months clinical assessment^1^** |  |
| Static two-point discrimination test | 58 (47.5) |
| Tactile gnosis using Shape/Texture Identification test | 70 (57.4) |
| Touch thresholds using Weinstein Enhanced Sensory Test monofilaments | 66 (54.1) |
| Cold intolerance | 73 (59.8) |
| Elliot neuroma score | 74 (60.7) |
| **3 months questionnaire^1^** |  |
| I-HaND | 74 (60.7) |
| EQ-5D-5L | 69 (56.6) |
| Patient’s Global Rating of Sensation | 7 (5.7) |
| **6 months questionnaire^1^** |  |
| EQ-5D-5L | 66 (54.1) |
| **12 months clinical assessment^1^** |  |
| Static two-point discrimination test | 23 (18.9) |
| Tactile gnosis using Shape/Texture Identification test | 27 (22.1) |
| Touch thresholds using Weinstein Enhanced Sensory Test monofilaments | 27 (22.1) |
| Cold intolerance | 28 (23.0) |
| Elliot neuroma score | 27 (22.1) |
| Patient’s Global Rating of Sensation | 46 (37.7) |
| **12 months questionnaire^1^** |  |
| I-HaND | 106 (86.9) |
| Hand Health Profile of the Patient Evaluation Measure | 102 (83.6) |
| EQ-5D-5L | 107 (87.7) |
| ^1^Summaries are n (%). | |

***Supplementary Table 7:*** *Withdrawals from follow-up split by treatment group*

|  | ***Suture repair (n=61)*** | ***Nerve alignment alone (n=61)*** |
| --- | --- | --- |
| *Withdrawals (before surgery)^1^* | *0* | *0* |
| *Withdrawals (between surgery and 6 weeks follow-up)^1^* | *1 (1.6)* | *2 (3.3)* |
| *Withdrawals (between 6 weeks and 3 months follow-up)^1^* | *0* | *0* |
| *Withdrawals (between 3 and 6 months follow-up)^1^* | *0* | *0* |
| *Withdrawals (between 6 and 12 months follow-up)^1^* | *1 (1.6)* | *1 (1.6)* |
| *Withdrawals (After 12 months follow-up)^1^* | *1 (1.6)* | *1 (1.6)* |

^1^Summaries are n (%).

| ***Supplementary Table 8:*** *Missing data in I-HaND (v2) scores by treatment group and timepoint*   \|  \| **Suture repair  (n=61)** \| **Nerve alignment alone  (n=61)** \| **Total  (n=122)** \| \| --- \| --- \| --- \| --- \| \| **6 weeks^1^** \| \| \| \| \| Missing \| 22 (36.07) \| 19 (31.15) \| 41 (33.61) \| \| **3 months^1^** \| \| \| \| \| Missing \| 29 (47.54) \| 19 (31.15) \| 48 (39.34) \| \| **12 months^1^** \| \| \| \| \| Missing \| 9 (14.75) \| 7 (11.48) \| 16 (13.11) \| \| ^1^Summaries are n (%). \| \| \| \|   ***Supplementary Table 9****: Results of the hand health profile by treatment groups – ITT population*   \|  \| **Suture repair  (n=61)** \| **Nerve alignment alone  (n=61)** \| **Adjusted diff (95% CI)^1^** \| **p-value** \| **Unadjusted diff (95% CI)^2^** \| **p-value** \| \| --- \| --- \| --- \| --- \| --- \| --- \| --- \| \| **The hand health profile** \| n=50, 34.93 (16.01) \| n=52, 32.27 (13.96) \| 4.03 (-3.36, 11.41) \| 0.26 \| 2.67 (-3.23, 8.56) \| 0.371 \| \| ^1^Linear regression model, adjusted for minimisation factors (critical or non-critical zone, associated tendon injury). Study site was accounted for using cluster robust variance. \| \| \| \| \| \| \| \| ^2^Unadjusted analysis was an independent t-test \| \| \| \| \| \| \|   ***Supplementary Table 10:*** *Results of the EQ-5D-5L index and the EQ-VAS by treatment groups – ITT population*   \| **Measure** \|  \| **Suture repair  (n=61)^1^** \| **Nerve alignment alone  (n=61)^1^** \| **Adjusted diff (95% CI)^2^** \| **p-value** \| **Unadjusted diff (95% CI)^3^** \| **p-value** \| \| --- \| --- \| --- \| --- \| --- \| --- \| --- \| --- \| \| **EQ-5D-5L** \| 6 weeks \| n=36, 0.68 (0.23) \| n=40, 0.75 (0.13) \| -0.09 (-0.17, -0.01) \| 0.033 \| -0.07 (-0.15, 0.01) \| 0.098 \| \|  \| 3 months \| n=32, 0.76 (0.11) \| n=37, 0.76 (0.19) \| 0.01 (-0.07, 0.09) \| 0.752 \| 0 (-0.08, 0.08) \| 0.999 \| \|  \| 6 months \| n=27, 0.77 (0.13) \| n=38, 0.82 (0.14) \| -0.04 (-0.09, 0.01) \| 0.084 \| -0.05 (-0.12, 0.02) \| 0.152 \| \|  \| 12 months \| n=53, 0.78 (0.27) \| n=54, 0.81 (0.17) \| -0.04 (-0.16, 0.07) \| 0.397 \| -0.02 (-0.11, 0.06) \| 0.605 \| \| **EQ-VAS** \| 6 weeks \| n=34, 70.94 (18.74) \| n=38, 76.13 (17.51) \| -6.02 (-17.86, 5.81) \| 0.291 \| -5.19 (-13.71, 3.33) \| 0.228 \| \|  \| 3 months \| n=32, 77.75 (12.29) \| n=39, 77.85 (16.81) \| -0.04 (-7.99, 7.92) \| 0.992 \| -0.1 (-7.21, 7.02) \| 0.979 \| \|  \| 6 months \| n=27, 75.96 (17.10) \| n=39, 82.28 (15.78) \| -5.85 (-16.21, 4.51) \| 0.242 \| -6.32 (-14.49, 1.85) \| 0.127 \| \|  \| 12 months \| n=53, 74.53 (18.63) \| n=54, 81.15 (16.76) \| -7.27 (-15.36, 0.83) \| 0.074 \| -6.62 (-13.41, 0.17) \| 0.056 \| \| ^1^Summaries are mean (SD) \| \| \| \| \| \| \| \| \| ^2^Linear regression model, adjusted for minimisation factors (critical or non-critical zone, associated tendon injury). Study site was accounted for using cluster robust variance. \| \| \| \| \| \| \| \|   ***Supplementary Table 11:*** *Results of Patient’s Global Rating of Sensation by treatment groups – ITT population*   \|  \| **Suture repair  (n=61)^1^** \| **Nerve alignment alone  (n=61)^1^** \| **Adjusted diff (95% CI)^2^** \| **p-value** \| **Unadjusted diff (95% CI)^3^** \| **p-value** \| \| --- \| --- \| --- \| --- \| --- \| --- \| --- \| \| **3 months** \| n=3, 3.67 (2.52) \| n=4, 4.75 (1.71) \| NA \| NA \| NA \| NA \| \| **12 months** \| n=21, 4.95 (2.16) \| n=25, 4.64 (2.66) \| 0.09 (-0.85, 1.02) \| 0.833 \| 0.31 (-1.15, 1.77) \| 0.668 \| \| ^1^Patient’s Global Rating of Sensation, Summaries are mean (SD) \| \| \| \| \| \| \| \| ^2^Linear regression model, adjusted for minimisation factors (critical or non-critical zone, associated tendon injury). Study site was accounted for using cluster robust variance. \| \| \| \| \| \| \| \| ^3^Unadjusted analysis was an independent t-test \| \| \| \| \| \| \|   ***Supplementary Table 12:*** *Results of Touch thresholds using Weinstein Enhanced Sensory Test (WEST) by treatment groups (3 levels)*   \|  \| **Suture repair  (n=61)** \| **Nerve alignment alone  (n=61)** \| **Adjusted diff (95% CI)^2^** \| **p-value** \| \| --- \| --- \| --- \| --- \| --- \| \| **3 months (3 levels)^1^** \| n=31 \| n=35 \| 0.82 (0.33, 2.04) \| 0.675 \| \| Black(1)/Red(2) \| 12 (38.71) \| 13 (37.14) \|  \|  \| \| Purple(3) \| 14 (45.16) \| 14 (40.00) \|  \|  \| \| Blue(4)/Green(5) \| 5 (16.13) \| 8 (22.86) \|  \|  \| \|  \|  \|  \| 4.66 (0.95, 26.62) \| 0.066 \| \| **12 months (3 levels)^1^** \| n=15 \| n=12 \|  \|  \| \| Black(1)/Red(2) \| 2 (13.33) \| 7 (58.33) \|  \|  \| \| Purple(3) \| 7 (46.67) \| 2 (16.67) \|  \|  \| \| Blue(4)/Green(5) \| 6 (40.00) \| 3 (25.00) \|  \|  \| \| ^1^Touch thresholds, Summaries are n (%). \| \| \| \| \| \| ^2^This is a post-hoc analysis in which touch thresholds were categorised into 3 levels instead of 5. Proportional Odds logistic regression model, adjusted for critical or non-critical zone and associated tendon injury \| \| \| \| \|   *Table 12* can be interpreted by saying that being in the Suture repair group is associated with an 18% lower odds of having being in a better level of the WEST score at 3 months, but is associated with a 366% higher odds of having a better WEST score at 12 months.  ***Supplementary Table 13:*** *Results of the Static two-point discrimination test (2PD) by treatment groups – ITT population*   \|  \| **Suture repair  (n=61)^1^** \| **Nerve alignment alone  (n=61)^1^** \| **Adjusted diff (95% CI)^2^** \| **p-value** \| **Unadjusted diff (95% CI)^3^** \| **p-value** \| \| --- \| --- \| --- \| --- \| --- \| --- \| --- \| \| **3 months** \| n=30, 9.10 (2.82) \| n=28, 10.79 (2.64) \| -1.83 (-3.64, -0.02) \| 0.048 \| -1.69 (-3.13, -0.25) \| 0.023 \| \| **12 months** \| n=11, 9.82 (2.71) \| n=12, 11.17 (2.44) \| -1.7 (-3.21, -0.2) \| 0.032 \| -1.35 (-3.58, 0.89) \| 0.224 \| \| ^1^The Static two-point discrimination test, summaries are mean (SD) \| \| \| \| \| \| \| \| ^2^Linear regression model, adjusted for minimisation factors (critical or non-critical zone, associated tendon injury). Study site was accounted for using cluster robust variance. \| \| \| \| \| \| \| \| ^3^Unadjusted analysis was an independent t-test \| \| \| \| \| \| \|   ***Supplementary Table 14:*** *Results of Tactile gnosis using Shape/Texture Identification test (STI) by treatment groups – ITT population*   \|  \| **Suture repair  (n=61)^1^** \| **Nerve alignment alone  (n=61)^1^** \| **Adjusted diff (95% CI)^2^** \| **p-value** \| **Unadjusted diff (95% CI)^3^** \| **p-value** \| \| --- \| --- \| --- \| --- \| --- \| --- \| --- \| \| **3 months** \| n=33, 1.94 (1.60) \| n=37, 2.46 (2.19) \| -0.46 (-1.18, 0.26) \| 0.191 \| -0.52 (-1.45, 0.41) \| 0.266 \| \| **12 months** \| n=15, 3.00 (1.77) \| n=12, 3.08 (2.07) \| -0.25 (-1.4, 0.89) \| 0.615 \| -0.08 (-1.6, 1.44) \| 0.911 \| \| ^1^STI test, summaries are mean (SD) \| \| \| \| \| \| \| \| ^2^Linear regression model, adjusted for minimisation factors (critical or non-critical zone, associated tendon injury). Study site was accounted for using cluster robust variance. \| \| \| \| \| \| \| \| ^3^Unadjusted analysis was an independent t-test \| \| \| \| \| \| \|   ***Supplementary Table 15:*** *Complications experienced and reviewed by health professional at 6 weeks post randomisation*   \| **Type** \| **Suture repair** \| **Nerve alignment alone** \| **Total** \| \| --- \| --- \| --- \| --- \| \| **Participants with complications at 6 weeks post randomisation** \| 26 \| 24 \| 50 \| \| Wound infection \| 1 \| 2 \| 3 \| \| Wound re-opening \| 2 \| 0 \| 2 \| \| Delayed wound healing \| 3 \| 2 \| 5 \| \| Scar sensitivity \| 14 \| 12 \| 26 \| \| Persistent ongoing pain \| 5 \| 2 \| 7 \| \| Other \| 3 \| 2 \| 5 \|   ***Supplementary Table 16:*** *Complications and further procedures in medical records at 3 months and 12 months post randomisation*   \|  \| **Suture repair (n=61)** \| **Nerve alignment alone (n=61)** \| \| --- \| --- \| --- \| \| **Participants with complications at 3 months post randomisation** \| 18 \| 20 \| \| Neuroma surgery \| 0 \| 0 \| \| Lack of sensitivity \| 6 \| 9 \| \| Hypersensitivity \| 1 \| 7 \| \| Cold intolerance \| 4 \| 3 \| \| Complex regional pain syndrome \| 0 \| 0 \| \| Flexor tendon re-rupture (capturing date of re-operation, if performed) \| 0 \| 1 \| \| Stiffness \| 7 \| 7 \| \| Swelling \| 5 \| 3 \| \| **Participants with complications at 12 months post randomisation** \| 13 \| 9 \| \| Neuroma surgery \| 0 \| 1 \| \| Potential neuroma^1^ \| 4 \| 1 \| \| Lack of sensitivity \| 5 \| 4 \| \| Hypersensitivity \| 1 \| 1 \| \| Cold intolerance \| 1 \| 1 \| \| Complex regional pain syndrome \| 0 \| 0 \| \| Flexor tendon re-rupture (capturing date of re-operation, if performed) \| 0 \| 1 \| \| Stiffness \| 2 \| 3 \| \| Swelling \| 5 \| 1 \| \|  \| \| \| \| ^1^Potential neuroma is defined at patients with a Total Elliot Score of 8 or more, or Total Elliot Score of 7 and at least one individual score of 3. This row was added post-hoc at the request of the investigators and therefore is not in the Statistical Analysis Plan. \| \| \|   ***Supplementary Table 17:*** *Summary of cold intolerance at 3 months and 12 months post randomisation by treatment groups – ITT population*   \|  \| **N** \| **Suture repair  (n=61)** \| **N** \| **Nerve alignment alone  (n=61)** \| \| --- \| --- \| --- \| --- \| --- \| \| **3 months** \| \| \|  \|  \| \| No discomfort or pain (0) \| 33 \| 10 (30.30) \| 40 \| 20 (50.00) \| \| Discomfort or pain that not interfere with usual activities (1) \| 33 \| 16 (48.48) \| 40 \| 13 (32.50) \| \| Discomfort or pain that interferes with some activities (2) \| 33 \| 7 (21.21) \| 40 \| 7 (17.50) \| \| **12 months** \| \| \|  \|  \| \| No discomfort or pain (0) \| 16 \| 4 (25.00) \| 12 \| 6 (50.00) \| \| Discomfort or pain that not interfere with usual activities (1) \| 16 \| 10 (62.50) \| 12 \| 6 (50.00) \| \| Discomfort or pain that interferes with some activities (2) \| 16 \| 2 (12.50) \| 12 \| 0 (0.00) \|   ***Supplementary Table 18:*** *Analysis of cold intolerance at 3 months and 12 months post randomisation by treatment groups – ITT population*   \|  \| \| **Suture repair^1^** \| \| **Nerve alignment alone^1^** \| \|  \| \| \| --- \| --- \| --- \| --- \| --- \| --- \| --- \| --- \| \|  \|  \| **0** \| **1 or 2** \| **0** \| **1 or 2** \| **Adjusted Odds Ratio (95% CI)^2^** \| **p-value** \| \| **Cold intolerance** \| **3 months** \| 10 (30.3) \| 23 (69.7) \| 20 (50) \| 20 (50) \| 0.46 (0.15, 1.29) \| 0.149 \| \|  \| **12 months** \| 9 (29) \| 22 (71) \| 18 (52.9) \| 16 (47.1) \| 0.44 (0.14, 1.32) \| 0.148 \| \| ^1^0 = No discomfort or pain, 1 = Discomfort or pain that does not interfere with usual activities, 2 = Discomfort or pain that interferes with some usual activities. Summaries are n(%). \| \| \| \| \| \| \| \| \| ^2^Logistic regression model, adjusting for minimisation factors (critical or non-critical zone, associated tendon injury and study site). The reference group was 0. \| \| \| \| \| \| \| \|   ***Supplementary Table 19:*** *Summary of Elliot score items at 3 months and 12 months post randomisation by treatment groups – ITT population*   \|  \| \| **Suture repair** \| \| \| \| **Nerve alignment alone** \| \| \| \| \| --- \| --- \| --- \| --- \| --- \| --- \| --- \| --- \| --- \| --- \| \|  \|  \| **0** \| **1** \| **2** \| **3** \| **0** \| **1** \| **2** \| **3** \| \| **Spontaneous pain –** \| **3 months** \| 22 \| 10 \| 1 \| 0 \| 33 \| 6 \| 2 \| 0 \| \| **basal** \| **12 months** \| 11 \| 3 \| 1 \| 0 \| 9 \| 2 \| 1 \| 0 \| \| **Spontaneous pain –** \| **3 months** \| 20 \| 7 \| 5 \| 1 \| 20 \| 11 \| 9 \| 1 \| \| **spikes** \| **12 months** \| 12 \| 0 \| 2 \| 1 \| 6 \| 5 \| 1 \| 0 \| \| **Pressure pain** \| **3 months** \| 15 \| 7 \| 8 \| 3 \| 25 \| 9 \| 7 \| 0 \| \|  \| **12 months** \| 7 \| 1 \| 6 \| 1 \| 8 \| 4 \| 0 \| 0 \| \| **Movement pain** \| **3 months** \| 16 \| 9 \| 8 \| 0 \| 23 \| 9 \| 9 \| 0 \| \|  \| **12 months** \| 8 \| 5 \| 2 \| 0 \| 9 \| 2 \| 1 \| 0 \| \| **Hypersensitivity** \| **3 months** \| 10 \| 12 \| 9 \| 2 \| 17 \| 11 \| 7 \| 6 \| \|  \| **12 months** \| 4 \| 5 \| 2 \| 4 \| 6 \| 3 \| 3 \| 0 \| \| Score 0: No pain, Score 1: Mild pain, Score 2: Moderate pain, Score 3: Severe pain \| \| \| \| \| \| \| \| \| \|   ***Supplementary Table 20:*** *Analysis of Elliot score at 3 months and 12 months post randomisation by treatment groups – ITT population*   \|  \| **Suture repair  (n=61)^1^** \| **Nerve alignment alone  (n=61)^1^** \| **Adjusted diff (95% CI)^2^** \| **p-value** \| **Unadjusted diff (95% CI)^3^** \| **p-value** \| \| --- \| --- \| --- \| --- \| --- \| --- \| --- \| \| **3 months** \| n=33, 3.79 (2.65) \| n=41, 3.29 (2.99) \| -0.52 (-2.31, 1.27) \| 0.537 \| -0.5 (-1.82, 0.83) \| 0.46 \| \| **12 months** \| n=15, 3.87 (3.81) \| n=12, 2.33 (2.53) \| -2.43 (-5.11, 0.24) \| 0.068 \| -1.53 (-4.18, 1.11) \| 0.243 \| \| ^1^Total Elliot Score, Summaries are mean (SD) \| \| \| \| \| \| \| \| ^2^Linear regression model, adjusted for minimisation factors (critical or non-critical zone, associated tendon injury). Study site was accounted for using cluster robust variance. \| \| \| \| \| \| \| \| ^3^Unadjusted analysis was an independent t-test \| \| \| \| \| \| \|   ***Supplementary Table 21:*** *Details of serious adverse events*   \| **Allocation** \| **Overall diagnosis** \| **Relatedness to intervention** \| \| --- \| --- \| --- \| \| **Control** \| Work related injury, dressing on right little finger caught in drill at work and sustained traumatic amputation. \| Not Related \| \| **Intervention** \| Persistent supraventricular tachycardia HR >130, ECG shows Atrial Flutter \| Not Related \| \| **Control** \| Patient presented at ACU with chest pain, swollen left knee and peripheral oedema. \| Not Related \| |
| --- | --- | --- | --- | --- | --- | --- | --- | --- | --- | --- | --- | --- | --- | --- | --- | --- | --- | --- | --- | --- | --- | --- | --- | --- | --- | --- | --- | --- | --- | --- | --- | --- | --- | --- | --- | --- | --- | --- | --- | --- | --- | --- | --- | --- | --- | --- | --- | --- | --- | --- | --- | --- | --- | --- | --- | --- | --- | --- | --- | --- | --- | --- | --- | --- | --- | --- | --- | --- | --- | --- | --- | --- | --- | --- | --- | --- | --- | --- | --- | --- | --- | --- | --- | --- | --- | --- | --- | --- | --- | --- | --- | --- | --- | --- | --- | --- | --- | --- | --- | --- | --- | --- | --- | --- | --- | --- | --- | --- | --- | --- | --- | --- | --- | --- | --- | --- | --- | --- | --- | --- | --- | --- | --- | --- | --- | --- | --- | --- | --- | --- | --- | --- | --- | --- | --- | --- | --- | --- | --- | --- | --- | --- | --- | --- | --- | --- | --- | --- | --- | --- | --- | --- | --- | --- | --- | --- | --- | --- | --- | --- | --- | --- | --- | --- | --- | --- | --- | --- | --- | --- | --- | --- | --- | --- | --- | --- | --- | --- | --- | --- | --- | --- | --- | --- | --- | --- | --- | --- | --- | --- | --- | --- | --- | --- | --- | --- | --- | --- | --- | --- | --- | --- | --- | --- | --- | --- | --- | --- | --- | --- | --- | --- | --- | --- | --- | --- | --- | --- | --- | --- | --- | --- | --- | --- | --- | --- | --- | --- | --- | --- | --- | --- | --- | --- | --- | --- | --- | --- | --- | --- | --- | --- | --- | --- | --- | --- | --- | --- | --- | --- | --- | --- | --- | --- | --- | --- | --- | --- | --- | --- | --- | --- | --- | --- | --- | --- | --- | --- | --- | --- | --- | --- | --- | --- | --- | --- | --- | --- | --- | --- | --- | --- | --- | --- | --- | --- | --- | --- | --- | --- | --- | --- | --- | --- | --- | --- | --- | --- | --- | --- | --- | --- | --- | --- | --- | --- | --- | --- | --- | --- | --- | --- | --- | --- | --- | --- | --- | --- | --- | --- | --- | --- | --- | --- | --- | --- | --- | --- | --- | --- | --- | --- | --- | --- | --- | --- | --- | --- | --- | --- | --- | --- | --- | --- | --- | --- | --- | --- | --- | --- | --- | --- | --- | --- | --- | --- | --- | --- | --- | --- | --- | --- | --- | --- | --- | --- | --- | --- | --- | --- | --- | --- | --- | --- | --- | --- | --- | --- | --- | --- | --- | --- | --- | --- | --- | --- | --- | --- | --- | --- | --- | --- | --- | --- | --- | --- | --- | --- | --- | --- | --- | --- | --- | --- | --- | --- | --- | --- | --- | --- | --- | --- | --- | --- | --- | --- | --- | --- | --- | --- | --- | --- | --- | --- | --- | --- | --- | --- | --- | --- | --- | --- | --- | --- | --- | --- | --- | --- | --- | --- | --- | --- | --- | --- | --- | --- | --- | --- | --- | --- | --- | --- | --- | --- | --- | --- | --- | --- | --- | --- | --- | --- | --- | --- | --- | --- | --- | --- | --- | --- | --- | --- | --- | --- | --- | --- | --- | --- | --- | --- | --- | --- | --- | --- | --- | --- | --- | --- | --- | --- | --- | --- | --- | --- | --- | --- | --- | --- | --- | --- | --- | --- | --- | --- | --- | --- | --- | --- | --- | --- | --- | --- | --- | --- | --- | --- | --- | --- | --- | --- | --- | --- | --- | --- | --- | --- | --- | --- | --- | --- | --- | --- | --- | --- | --- | --- | --- | --- | --- | --- | --- | --- | --- | --- | --- | --- | --- | --- | --- | --- | --- | --- | --- | --- | --- | --- | --- | --- | --- | --- | --- | --- | --- | --- | --- | --- | --- | --- | --- | --- | --- | --- | --- | --- | --- | --- | --- | --- | --- | --- | --- | --- | --- | --- | --- | --- | --- | --- | --- | --- | --- | --- | --- | --- | --- | --- | --- | --- | --- | --- | --- | --- | --- | --- | --- | --- | --- | --- | --- | --- | --- | --- | --- | --- | --- | --- | --- | --- | --- | --- | --- | --- | --- | --- | --- | --- | --- | --- | --- | --- | --- | --- | --- | --- | --- | --- | --- | --- | --- | --- | --- | --- | --- | --- | --- | --- | --- | --- | --- | --- | --- | --- | --- | --- | --- | --- | --- | --- | --- | --- | --- | --- | --- | --- | --- | --- | --- | --- | --- | --- | --- | --- | --- | --- | --- | --- | --- | --- | --- | --- | --- | --- | --- | --- | --- | --- | --- | --- | --- | --- | --- | --- | --- | --- | --- | --- | --- | --- | --- | --- | --- | --- | --- | --- | --- | --- | --- | --- | --- |

**Supplementary Material 1 - NEON Study Team**

**Chief Investigator(s)**: Abhilash Jain (Oxford), David Beard (Oxford), Dominic Power (Birmingham)

**Trial co-investigators:** Justin Wormald (Oxford), Matthew D. Gardiner (Oxford), Cristina Jerosch-Herold (Norwich), Jonathan Cook (Oxford), Rafael Pinedo Villanueva (Oxford), Cushla Cooper (Oxford), Dominic Power (Birmingham).

The study was managed by the Surgical Intervention Trials Unit (SITU) in collaboration with the Oxford Clinical Trials Research Unit (OCTRU).

**Trial project/data management/oversight**: Scott Parsons, Molly Glaze, Anjali Shah, David Smith, Heidi Fletcher, Loretta Davies.

**Trial administration**: Akiko Greshon Charlotte Drodge, Jacob Moyse

**Trial statisticians**: Naomi Vides, Ariel Wang, Beverly Shirkey

**Health economists**: Gianluca Fabiano, Sophie Cole, Spyros Kolovos

We are grateful to the participants and research teams at collaborating hospital sites, without whom the trial would not have been possible.

**Participating sites and Principal Investigator (PI):**

Hampshire Hospitals NHS Foundation Trust – PI: Duncan Avis

Mid and South Essex NHS Foundation – PI: Adam Sierakowski

University Hospitals of Derby and Burton NHS Foundation Trust - PI: Chris Bainbridge

Imperial College Healthcare NHS Trust - PI: Kshemendra Senarath-Yapa

The Newcastle Upon Tyne Hospitals NHS Foundation Trust – PI: Susan Stephenson

Oxford University Hospitals NHS Foundation Trust – PI: Dominic Furniss

Queen Victoria Hospital NHS Foundation Trust – PI: Robert Pearl

Royal Cornwall Hospitals NHS Trust – PI: Rebecca Dunlop

Buckinghamshire Healthcare NHS Trust – PI: James Chan

University Hospitals Coventry & Warwickshire NHS Trust – PI: Clare Langley

Norfolk and Norwich University Hospitals NHS Foundation Trust – PI: Nick Sheppard

South Tees Hospitals NHS Foundation Trust – PI: Sarah Kettle

Frimley Health NHS Foundation Trust – PI: Richard Baker

Royal Devon University Healthcare NHS Foundation Trust – PI: Oliver Stone

We would like to thank the external members of the Trial Steering Committee and Data Monitoring

Committee for their advice and support for the project:

**Trial Steering Committee**

Professor Anne Schilder (Chairperson),Professor Hema Mistry, Mr Aadil Khan, Mr Andrew Elders, Professor Alison Hammond, Mr Oli O’Neill, Ms Kelly Richardson (Patient Representative)

**Data Monitoring Committee**

Professor Chris Metcalfe – (Chairperson), Mrs Mary-Clare Miller, Professor Fiona Cramp
